# Quantifying the role of antecedent Southwestern Indian Ocean capacitance on the summer monsoon rainfall variability over homogeneous regions of India

**DOI:** 10.1038/s41598-023-32840-w

**Published:** 2023-04-05

**Authors:** Venugopal Thandlam, Hasibur Rahaman, Anna Rutgersson, Erik Sahlee, M. Ravichandran, S. S. V. S. Ramakrishna

**Affiliations:** 1 Air, Water and Landscape Science (LUVAL), Department of Earth Sciences, Uppsala University, Uppsala, Sweden; 2 The Center for Environment and Development Studies Research Forum, Uppsala University, Uppsala, Sweden; 3 Centre of Natural Hazards and Disaster Science, Uppsala University, Uppsala, Sweden; 4 ESSO-Indian National Centre for Ocean Information Services (INCOIS), Ministry of Earth Sciences, Hyderabad, India; 5 Earth System Science Organization, Ministry of Earth Sciences, New Delhi, India; 6 Department of Meteorology and Oceanography, Andhra University, Visakhapatnam, India

**Keywords:** Ocean sciences, Climate sciences, Atmospheric science, Ocean sciences

## Abstract

The role of ocean variability is at a focal point in improving the weather and climate forecasts at different spatial and temporal scales. We study the effect of antecedent southwestern Indian Ocean mean sea level anomaly (MSLA) and sea surface temperature anomalies (SSTA) as a proxy to upper ocean heat capacitance on all India summer monsoon rainfall (AISMR) during 1993–2019. SSTA and MSLA over the southwestern Indian Ocean (SWIO) have been influenced by El Niño-Southern Oscillation (ENSO), the impact of ENSO-induced SWIO variability was low on rainfall variability over several homogeneous regions. Rainfall over northeast (NE) and North India (EI) has been modulated by ENSO-induced SSTA and MSLA over SWIO, thus effecting the total AISMR magnitude. The ENSO-induced changes in heat capacitance (SSTA and MSLA) over SWIO during antecedent months has less impact on west coast of India, central India and North India (NI) rainfall variability. The long-term trend in pre-monsoonal SSTA and MSLA over SWIO shows decreasing rainfall trend over NI, NE, and EI in the recent time. Furthermore, the cooler (warmer) anomaly over the western Indian Ocean affects rainfall variability adversely (favourably) due to the reversal of the wind pattern during the pre-monsoon period. While SSTA and MSLA are increasing in the SWIO, large-scale variability of these parameters during preceding winter and pre-monsoon months combined with surface winds could impact the inter-annual AISMR variability over homogeneous regions of India. Similarly, from an oceanic perspective, the antecedent heat capacitance over SWIO on an inter-annual time scale has been the key to the extreme monsoon rainfall variability.

## Introduction

The Asian monsoon circulation influences most of the tropics and subtropics of the eastern hemisphere and more than 60% of the Earth’s population^1,2^. Monsoon variations, mainly unanticipated, impart significant economic and social damages and consequences. It’s failure often brings famine to affected regions, and strong monsoon years can result in devastating floods^3^. Meanwhile, recent rapid changes in the global climate and warming temperatures increase the demand for local and regional weather forecasting and analysis to improve the accuracy of seasonal forecasting of extreme events such as droughts and floods. An accurate long-range seasonal and intra-seasonal predictions of monsoon rainfall can improve planning to act on monsoon’s adverse impacts and benefits^1,4^. Hence, a better understanding of the monsoon cycle is clearly of scientific and social value. Yet, all India summer monsoon rainfall (AISMR) variability on the inter-annual and intraseasonal time scale has puzzled the scientific community due to its complex and regional heterogenity^5,6^. With innovative technological advancement and many years of research, most dynamical and statistical models still fail to predict the seasonal and intraseasonal AISMR variability and associated extremes with reasonable accuracy^7–9^. This could be due to the unpredictable variability within the AISMR and the lack of understanding of the ocean’s role in AISMR variability. This implies that the persistent ambiguity on the impact of the slowly responding ocean surface to the extreme atmosphere–ocean coupled phenomena such as El Niño-Southern Oscillation (ENSO) during the antecedent months and its imprint on the rainfall variability of the following year^10–12^. Many previous studies have shown the role of ENSO in the Indian Ocean and on seasonal AISMR. But it’s the effect on different parts of the Indian mainland i,e., homogeneous regions, has not been examined yet. Hence, this study focused on addressing this aspect.

Blanford^13^ was the first to attempt a forecast of the seasonal AISMR following the disastrous famine of 1877. The study was based on the hypotheses that the varying extent and thickness of the Himalayan snow exercise a tremendous and prolonged influence on the climatic conditions and weather of the plains of northwest India^13^. Since then, much research has been done on predicting AISMR^14–18^. Shukla^19^ suggested that colder sea surface temperature anomalies (SSTA) over the western Arabian Sea and Somali coast may cause a reduction in AISMR over India and adjoining areas. AISMR is significantly positively correlated with Indian Ocean SSTA and moisture flux transport in the preceding winter and spring seasons on the time scale of the Tropical Biennial Oscillation^20^. Several empirical studies show a strong positive correlation of Arabian Sea SSTA averaged over March, April, and May with AISMR^21–23^. Harzallah and Sadourny^24^ investigated the lag-lead relationships of global SSTA and the AISMR Index from 1950 to 90. In the fall and winter preceding a strong monsoon, they found positive SSTA in the Indian Ocean, especially in the Arabian Sea. Vecchi and Harisson^25^ have shown that warm SSTA over the western Arabian Sea at the AISMR onset is associated with increased Western Ghat rainfall along the west coast of India, while cool SSTA off Java and Sumatra is associated with increased precipitation over the Ganges-Mahanadi basin. AISMR also has a significant and positive correlation with latent heat and momentum flux induced by SSTA during antecedent winter over the Arabian Sea, Bay of Bengal, and the South China Sea^26^. Many other studies also highlighted the positive correlations between pre-monsoon months’ SSTA over the western Arabian Sea and the southwestern Indian Ocean (SWIO)^27^ during AISMR^28–31^. Kothawale et al.^27,32^ have found that November SSTA over the Arabian Sea, Bay of Bengal, and equatorial South Indian Ocean is strongly correlated with AISMR during 1971–2002. Out of five homogeneous regions in India, monsoon rainfall over the West Coast of India (WCI) showed a significant relationship with Arabian SSTA of the preceding spring and winter months^32^. At the same time, Bay of Bengal SSTA in the preceding November and March and equatorial South Indian Ocean SSTAs of the preceding November, February, and March showed a substantial impact on WCI rainfall variability^20,33^. Thus, many past studies concentrated only on relationships between pre-monsoon SSTA over the Indian Ocean region and AISMR seasonal variability and forecasting^28–31^. As the AISMR is a coupled atmosphere–ocean phenomenon, the role of air-sea interactions over the southwestern and equatorial Indian Ocean is the key to the better understanding and forecasting of its magnitude and variability over India^34–36^. Shankar and Shetye^37^ have suggested that the interdecadal variability of sea level at Mumbai mimicked the variability in rainfall over the Indian subcontinent. Similarly, ocean mean temperature, representing the upper ocean heat energy over the SWIO during pre-monsoon months of the same year, shows a strong statistical relationship with the AISMR^34^. Venugopal et al.^35^ statistically illustrated that ocean mean temperature during January, February, and March over the SWIO could be a better ocean parameter for AISMR seasonal forecasting and variability during normal synoptic conditions. Thus, a few studies have focused on the relationship between sea level variations and subsurface ocean variability over the Indian Ocean region and AISMR^34,35^. Some of these studies stressed the use of new statistical techniques and parameters, such as the strength of the winds, ocean heat content and ocean-integrated subsurface temperatures in the seasonal forecasting^35,36^. Distinct impacts of short- and long-time fluctuations of the Indian Ocean surface wind fields, particularly over the SWIO, also led to changes in the rainfall over homogeneous regions of India^38^.

Monthly, seasonal, and regional rainfall intensities contribute to the total magnitude of annual AISMR. But rainfall during July–August contributes to the total extent of seasonal rain, regardless of the strength (strong or weak) of the summer monsoon^39^. The strength of the monsoon and intraseasonal variability (MISO) depends on the prevailing synoptic conditions and intraseasonal variability of atmospheric and ocean parameters over the tropics, particularly in the tropical Indian and Pacific Oceans^40–44^. Recently Saha et al.^45^ have shown that the synoptic variability, previously considered as noise, is predictable and has maximum contribution to the seasonal AISMR anomaly. Therefore, AISMR is a highly predictable system on a seasonal time scale. These synoptic activities and MISO, which are smaller in magnitude and affect the intensity of rainfall, are found to be associated with the planetary scale circulations like Madden–Julian Oscillation (MJO), ENSO, Indian Ocean Dipole, Pacific Decadal Oscillation and North Atlantic Oscillation^46–51^. Thus, the predictability of AISMR also lies on the planetary scale events, which evolve on a longer time scale and may leave their signature and impacts on the smaller-scale events to persist for a longer time^45^.

Though several studies found a relationship between summer monsoon and the upper ocean parameters over the SWIO concerning preceding months of the same year, none has focused on the relationship between rainfall over homogeneous regions of the Indian landmass and the SWIO. The question here is, does the Indian Ocean variability affect the entire Indian mainland AISMR or not? and how does the ENSO would impact this relationship? To the best of our knowledge, no studies are available as of now about how the Indian Ocean variability impacts the different sub-divisions of India, popularly known as homogeneous regions. The high ocean heat capacitance could hold the signature of planetary-scale events to persist for an extended period, thus impacting the synoptic conditions in the following years. Hence, studying the role of antecedent upper ocean capacitance over the SWIO on AISMR and other homogeneous regions with and without the impact of planetary-scale events like ENSO could provide more insights into the influence of the air-sea interactions on synoptic conditions over this region. Furthermore, exploring the effect of the SWIO capacitance on homogeneous rainfall regions of India could give a more localized glance at physical processes and altering air-sea interactions due to recent climate change over these regions leading to changes in the frequencies and intensities of extreme floods and droughts^52,53^. Also, quantifying the set of atmospheric and ocean parameters in seasonal numerical weather forecasting systems such as ECMWF’s new long-range forecasting system SEAS5 is a high priority to improve the forecast precision. Yet, a wide range of conflicting results can be found describing the relationship between Indian Ocean SSTA and Indian continental rainfall anomalies, which may partly arise because of uncertainties in our knowledge of Indian Ocean SSTA^25^. Much of the Indian Ocean was not well observed during the last century either by satellite observations or automated profiling floats such as ARGO. There were only observations from ships and drifting buoys^54,55^. Many studies have noted that the statistical relationship between monsoon variability and upper ocean parameters can be different in recent decades than in earlier decades^3,56–58^. The advent of satellite altimetry and microwave techniques to measure sea level anomaly (SLA) and SSTA, respectively, have provided data sets with better spatial and temporal coverage over the Indian Ocean region and contributed to the betterment of this statistical relationship in recent times.

On the other hand, sub-surface temperature and salinity profile data from scattered XBT, CTD, ARGO, and buoy locations were previously available with spatial and temporal sampling errors. However, ARGO has recently made revolutions in this aspect by measuring temperature/salinity (T/S) profile data since 2001 over the global ocean, including the Indian Ocean from 2003^59,60^. Although the spatial distribution pattern of ARGO profiling floats was sparse during the initial phase, it has reached its objective to have at least one profile in a 3 × 3-degree domain in 2008 over the global ocean, including the Indian Ocean^61^. The other most reliable subsurface information comes from the sea level measurement since it shows the mirror image of subsurface variation on the surface. This data has been available from satellite altimetry with high spatial resolutions since 1992.

In this study, we use these high spatial resolution datasets during 1993–2019 to determine the relationship between AISMR and MSLA and SSTA before and after removing the Nino3.4 SSTA effect. A robust and distinct relationship with these parameters in the Indian Ocean has been found in recent years, especially after 2001^29^. We examined the role of these parameters over the SWIO in the rainfall variability of different homogeneous regions of India. “Data” section describes the datasets used, and “Methodology” section describes the methodology adopted in the study. Subsequently, “Results and discussions” section describes the results and discussions. Finally, the conclusions are summarized in “Climatic effect of the Indian Ocean on AISMR” section.

## Data

The high-resolution (0.25 × 0.25) blended analysis of daily Optimum Interpolation SST (OISSTv2.1, also known as Reynolds’ SST) obtained from the National Oceanic and Atmospheric Administration (NOAA) during 1993–2019^62^ has been used in the study. In addition, we used delayed-time (reprocessed) daily sea level anomalies (SLA) data for the 1993–2019 period with a spatial resolution of 0.25°, obtained from Copernicus Marine Environment Monitoring Service (CMEMS)^63^. This product is obtained by combining fully processed data from various altimeter missions (Topex/Poseidon, ERS-1/2, Jason-1, Envisat and OSTM/Jason-2). The daily AISMR data has been extracted from the high-resolution (0.25 × 0.25) daily rainfall data constructed from more than 7000 rain gauge stations around India during the study period^64,65^. The AISMR data used in the study show a lower seasonal magnitude of rainfall than the data from Rajeevan et al.^65^,which has the 1° × 1° resolution (Figure not shown). The bias could be due to the annual changes in the number of rain gauge stations used in constructing the data. Despite its lower magnitude, the dataset has been used in the study owing to its higher spatial resolution and our aim of studying the rainfall variability rather the magnitude. Based on the rainfall intensity and variability, homogeneous regions of AISMR are broadly divided into north India (NI), east India (EI), northeast India (NE), central India (CI), and WCI^37,66^. More details on selected regions are provided in Table 1. Similarly, the Nino3.4 (170° W–120° W and 5° S–5° N) region’s monthly SSTA indices for ENSO were obtained from the Royal Netherlands Meteorological Institute climate explorer^67^. In addition, monthly surface zonal and meridional wind anomalies constructed from ECMWF-ERA5 with the 0.25 × 0.25-degree resolution have been used in the composite analysis^68^. Table 2 shows the details of the data used in the study.

**Table 1:** Details of homogeneous rainfall regions considered in the study.

Region name	Latitude–Longitude area	Short name	Seasonal mean during 1993–2019 (mm)	Seasonal standard deviation during 1993–2019 (mm)	Coefficient of variation
All India Summer monsoon rainfall	Indian mainland	AISMR	850.7	68.3	0.08
West coast of India	72 E–78 E; 8 N–22 N	WCI	2305	346	0.15
North India	74.5 E–84.5 E; 26.5 N–33.5 N	NI	687	111.9	0.16
East India	84.5 E–88.5 E; 18 N–28.5 N	EI	1137	135.7	0.12
Northeast India	88.5 E–97 E; 22 N–30 N	NE	1484	211	0.14
Central India	74.5 E–84.5 E; 16.5 N–26.5 N	CI	877.7	106.9	0.12

**Table 2:** Details of data used in the study.

S. No	Parameter/index	Grid spacing	Temporal resolution	Data source	Study region	References
1	All Indian Summer Monsoon Rainfall (AISMR)	0.25° × 0.25°	Daily	1	Indian mainland	Pai et al.^64^
2	Sea surface temperature	0.25° × 0.25°	Daily	2	40° E–100° E, 30° S–30° N	Reynolds et al.^62^
3	Sea level anomaly	0.25° × 0.25°	Daily	3	40° E–100° E, 30° S–30°N	Rosmorduc et al.^63^
4	Nino3.4 (EL NIÑO) index	1° × 1°	Monthly	4	5° N–5° S, 170° W–120° W	Rayner et al.^67^
5	Zonal and meridional wind components	0.25° × 0.25°	Monthly	5	40° E–100° E, 30° S–30° N	Hersbach et al. ^68^

Among the five homogeneous regions considered in the study, WCI and NE have the highest seasonal rainfall and standard deviation (Table 1), followed by EI, CI, and NI. Hence, a seasonal departure from average rainfall over WCI and NE could influence the total AISMR magnitude. On the other hand, being a region with a low standard deviation, the CI receives consistent seasonal rainfall and covers a larger area than any other homogeneous region considered in the study. Thus, this region is also key for the absolute magnitude of the AISMR.

The availability of these data sets is different over time. However, for uniformity, the time period for all the datasets used was 1993–2019.

Data source references: 1-India Meteorological Department; 2-National Oceanic and Atmospheric Administration; 3-Copernicus Marine Environment Monitoring Service; 4-Royal Netherlands Meteorological Institute; 5-Copernicus Climate Data Store (CDS).

## Methodology

### Identifying anomalous monsoon years

**Figure 1:**
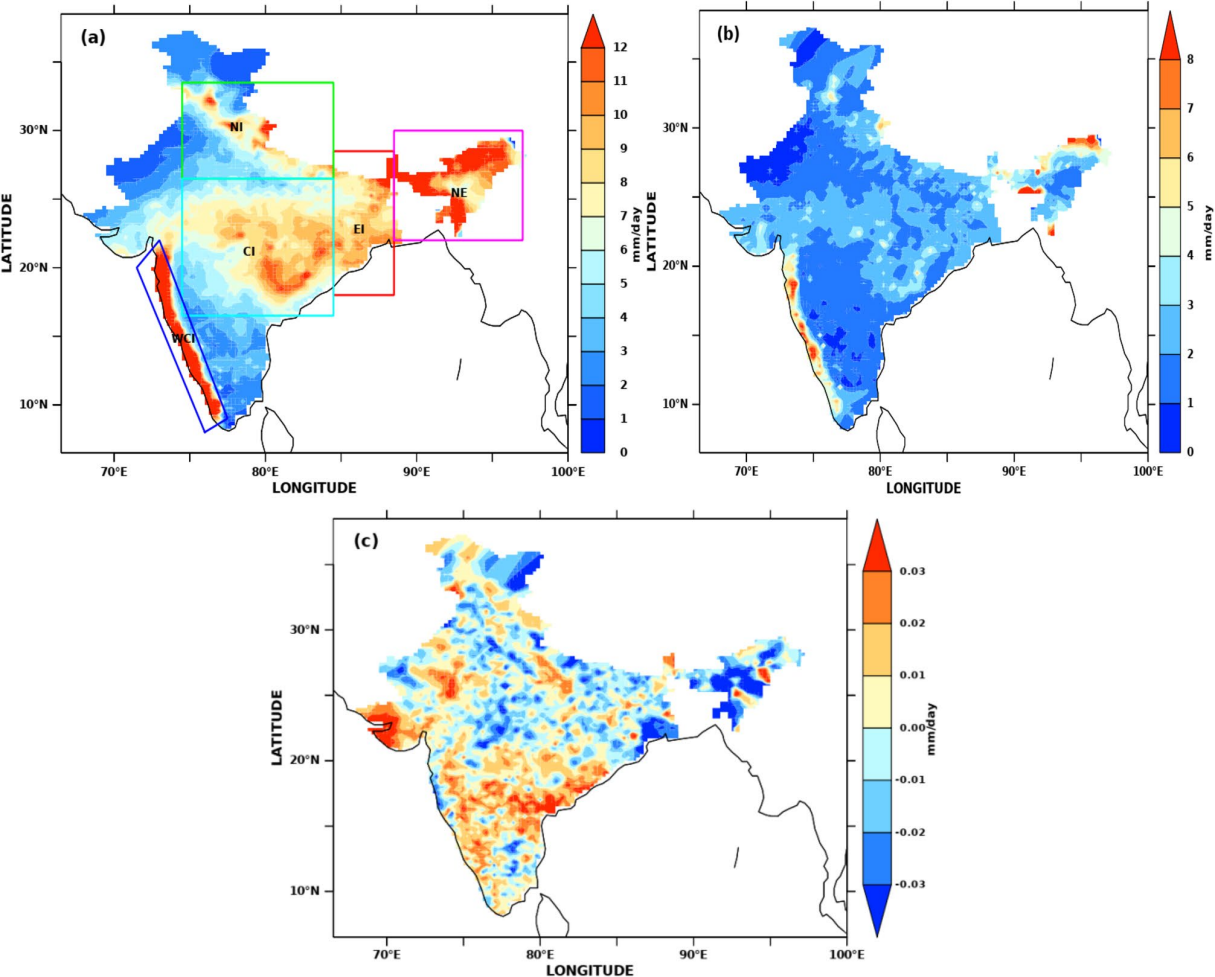
(**a**) AISMR (June–September) climatology (mm/day) during 1993–2019 in colour. Homogeneous rainfall regions are marked in different boxes: NI-North India, CI-Central India, WCI-West coast of India, NE-Northeast India, and EI-East India. (**b**) spatial pattern of daily AISMR standard deviation for JJAS during 1993–2019 (**c**) spatial distribution of mean SAI during 1993–2019. The figure is generated using PyFerret v7.63.

Krishnamurthy and Shukla^69^ have shown that the interannual variability of the Indian monsoon rainfall can be considered as a linear combination of a large-scale persistent seasonal mean component and a statistical average of intraseasonal variability in the gridded rainfall data. SSTA influences this large-scale persistent component. Therefore, success in long-range forecasting depends on accurate quantitative estimates of the externally forced component due to the intrinsically unpredictable intraseasonal component. Thus, our study looked at two external forcing fields, SSTA and MSLA, over the Indian Ocean in relation to the AISMR standard anomaly index (SAI). The monthly anomaly fields for MSLA and SSTA were constructed by computing monthly SLA and monthly SST and then subtracting it from monthly climatology during 1993–2019. Figure 1a,b shows the spatial pattern of AISMR seasonal mean (climatology) and standard deviation, respectively, during 1993–2019. WCI, NE, and CI contribute significantly to the total magnitude of AISMR, followed by EI and NI. The all-India mean is ~ 7 mm/day which is similar to the values reported by Rahman et al. ^70^ and Saha et al.^71^. Regions with a standard deviation less than 4 mm/day in southern peninsular India, the northernmost part of India and northwestern India are in shadow regions. These regions receive less rainfall during the summer monsoon. Thus, these regions' contribution to AISMR magnitude is minimal and not regarded as homogeneous regions.

To identify the years of anomalous monsoon rainfall, we adopted Mooley and Parthasarathy’s^72^ procedure. We use the four months (June–September) average of each year as a single point representing a season and thus used 27 years of data to compute the seasonal SAI. Accordingly, the SAI for AISMR and the homogeneous regions of summer monsoon rainfall (June–September) were computed in the following way,

1 $${\upsigma }_{i}= \frac{{(R}_{i}-{R}_{m})}{{\sigma }_{m}}$$

where “σ_i_” is the SAI of rainfall during the year i; “R_i_” is the monsoon rainfall (June–September mean) of the year i, with i = 1993…2019. “R_m_” is the mean seasonal (June–September) rainfall of 27 years, and “σ_m_” is the standard deviation of the AISMR (June–September) rainfall during 1993–2019 (i.e., for 27 years). These SAI values categorize monsoon rainfall as deficient or excess using the following thresholds.

$${\text{Deficit}} = \sigma_{i} < - 0.2$$

$${\text{Excess}} = \sigma_{i} > 0.2$$

$${\text{Normal}} = - 0.2 \le \sigma_{i} \le 0.2$$

Table 3 shows the details of normal, deficit and excess monsoon years during the study period. Figure 1c shows the spatial distribution of mean SAI over 1993–2019 with larger values over southern peninsular and western India, where the mean seasonal rainfall is less. We use SAI instead of mean because it represents the most appropriate measure of AISMR variability. In contrast, the mean is the best measure of the centre and can be used only when the distribution of AISMR data is normal.

**Table 3:** Details of normal, deficit and excess monsoon years during 1993–2019.

Excess years (σ_i_)	Deficit years (σ_i_)	Normal years (σ_i_)	
1994 (0.36)	2000 (− 0.22)	1993 (0.09)	2005 (− 0.01)
2007 (0.30)	2002 (− 0.56)	1995 (0.14)	2006 (0.17)
2011 (0.28)	2004 (− 0.30)	1996 (0.02)	2008 (0.09)
2013 (0.30)	2009 (− 0.46)	1997 (0.01)	2010 (0.18)
2019 (0.43)	2014 (− 0.28)	1998 (0.12)	2012 (− 0.05)
	2015 (− 0.30)	1999 (− 0.15)	2016 (0.05)
	2018 (− 0.20)	2001 (− 0.17)	2017 (0.01)
		2003 (0.17)	

Normal summer monsoon rains are present in fifteen (15) out of twenty-seven (27) years (55%), with SAI values between − 0.2 and 0.2. On the other hand, seven (7, 26%) years received deficit monsoon, and five (5) years received excess summer monsoon rains (19%) in the study period. No deficit monsoon years were found in the first decade (1993–1999), but there was one year with an excess monsoon in 1994 and six (6) normal monsoon years. The second decade (2000–2009) is more dynamic, with four (4) deficit years, one excess (2007) and five (5) normal monsoon years. With the changing climate, the recent decade (2010–2019) witnessed the highest number of excess monsoon years i,e., three (3) and the lowest number in four (4) normal monsoon years, with three (3) years being deficit monsoon years. Thus, in recent times monsoon rains show a significant deviation from normal rainfall magnitude and thus causing monsoon prediction to be more volatile and challenging.

### Removal of ENSO effect on the relationship between Indian Ocean SSTA, MSLA and AISMR SAI

To remove the ENSO effect on the relationship between AISMR (and all homogeneous regions) and SSTA/MSLA, we adopted the procedure of Kothawale et al.^32^. The influence of Nino3.4 is removed with the following equation.

2 $${r}_{123}=\frac{({r}_{12}-{r}_{13}*{r}_{23})}{\surd (\left(1-{r}_{13}^{2}\right)*\left(1-{r}_{23}^{2}\right))}$$

where r_123_: Correlation between SSTA/MSLA over the Indian Ocean and AISMR (including homogeneous regions) SAI excluding the effect of Nino3.4. r_12_: Lagged correlation between SSTA/MSLA over the Indian Ocean and AISMR (including homogeneous regions) SAI. r_23_: Lagged correlation between AISMR (including homogeneous regions) SAI and Nino3.4 SSTA indices. r_13_: Simultaneous correlation between Indian Ocean SSTA/MSLA and Nino3.4 SSTA indices

All data (rainfall, SSTA, MSLA and wind) are detrended before performing the correlation and composite analysis. A comparison between normal and detrended data for all these variables is shown in supplementary Fig. S1. An illustration showing the methodology implemented is also shown in supplementary Fig. S2.

### Ethics approval

The manuscript has not been submitted to more than one journal for simultaneous consideration. The manuscript has not been published previously (partly or in full) unless the new work concerns an expansion of previous work. Our study is not split up into several parts to increase the number of submissions and submitted to various journals or to one journal over time. No data has been fabricated or manipulated (including images) to support our conclusions. No data or text by others are presented as if they were our own (“plagiarism”).

## Results and discussions

### Effect of NINO3.4 on Indian Ocean SSTA and MSLA

**Figure 2:**
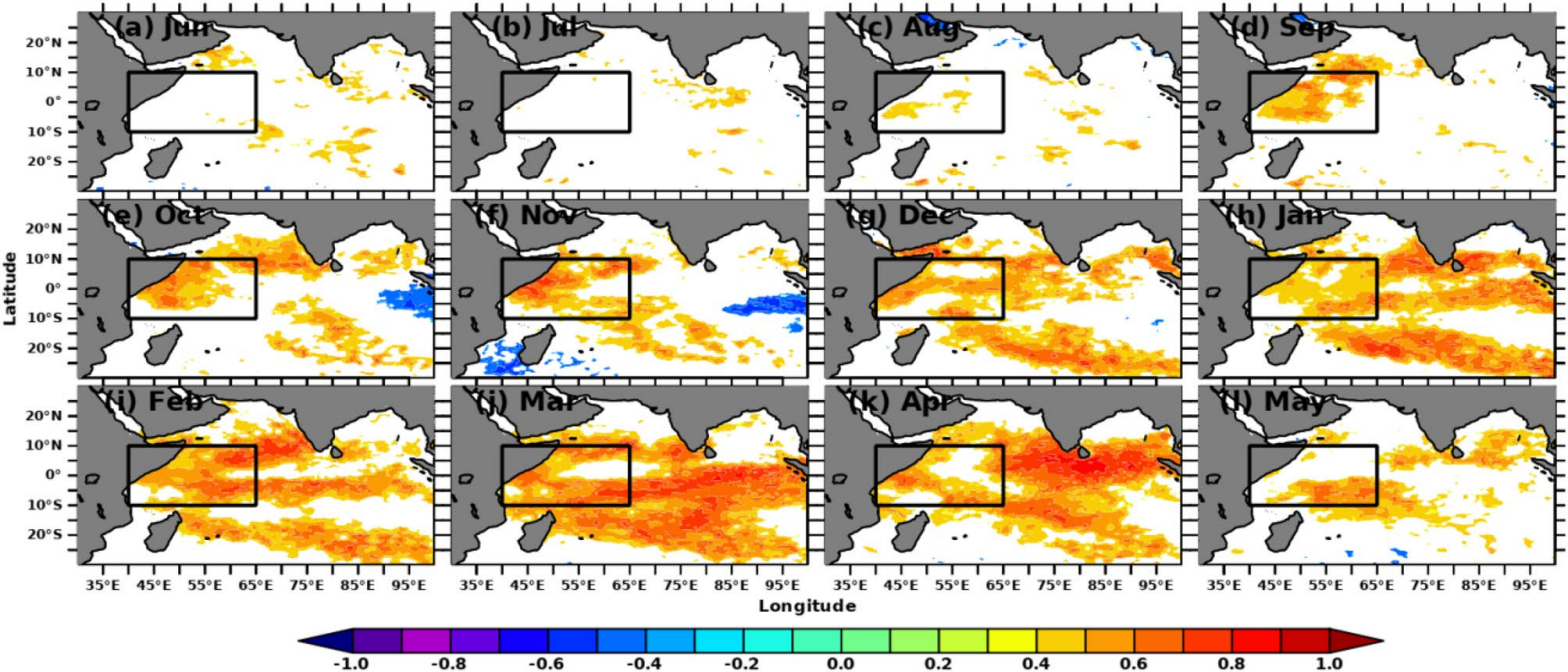
Simultaneous monthly correlations between detrended Indian Ocean SSTA and Nino3.4 SSTA indices. Areas of correlations at 95% significance are shown. The figure is generated using PyFerret v7.63.

**Figure 3:**
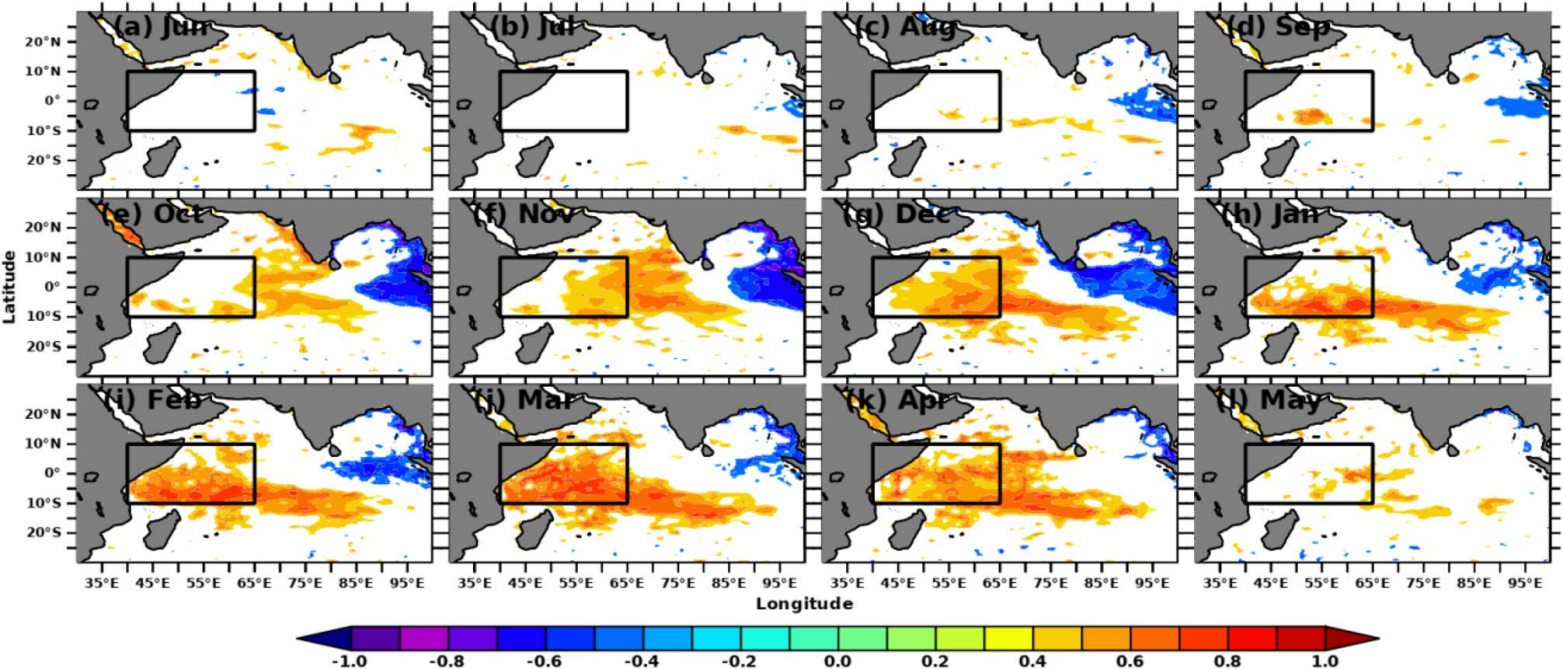
Simultaneous monthly correlations between detrended Indian Ocean MSLA and Nino3.4 SSTA indices. Areas of correlations at 95% significance are shown. The figure is generated using PyFerret v7.63.

The simultaneous monthly correlations of Indian Ocean SSTA and MSLA with Nino3.4 SSTA indices are shown in Figs. 2 and 3, respectively. Nino3.4 SSTA indices measure the occurrence and intensity of ENSO. While each ENSO event is unique, the intensity of a typical ENSO event would last for 9–12 months, with its lifecycle being about 4 years^73,74^. A typical ENSO starts during boreal summer (Mar-Jun) and peaks during boreal winter (Dec–Feb)^75,76^. Hence, we computed the spatial correlation in the Indian Ocean concerning Nino3.4 SSTA from previous years’ June month. Starting from September, both SSTA and MSLA over SWIO show a positive correlation (r > 0.5) with Nino3.4, which is significant at a 95% confidence level. The spatial extent of the significant correlations extends in the subsequent months and reaches its maximum during Mar-April. This result suggests that the Nino3.4 SST impact the SWIO and surrounding ocean regions during the preceding winter and spring with strong intraseasonal to interannual variability. Previous studies show that ENSO impacts SWIO through the Rossby wave, which propagates from the eastern to western Indian Ocean^77–79^. With the large heat capacitance of ocean waters, the SWIO may continue to affect the air-sea interactions over this region during later months and thus alter the rainfall in the following summer monsoon^80,81^. Hence, improved monsoon forecasting would be possible with a deeper understanding of the role of SSTA and MSLA over the SWIO on the homogeneous rainfall regions of the Indian mainland.

### Indian Ocean SSTA and MSLA variability

**Figure 4:**
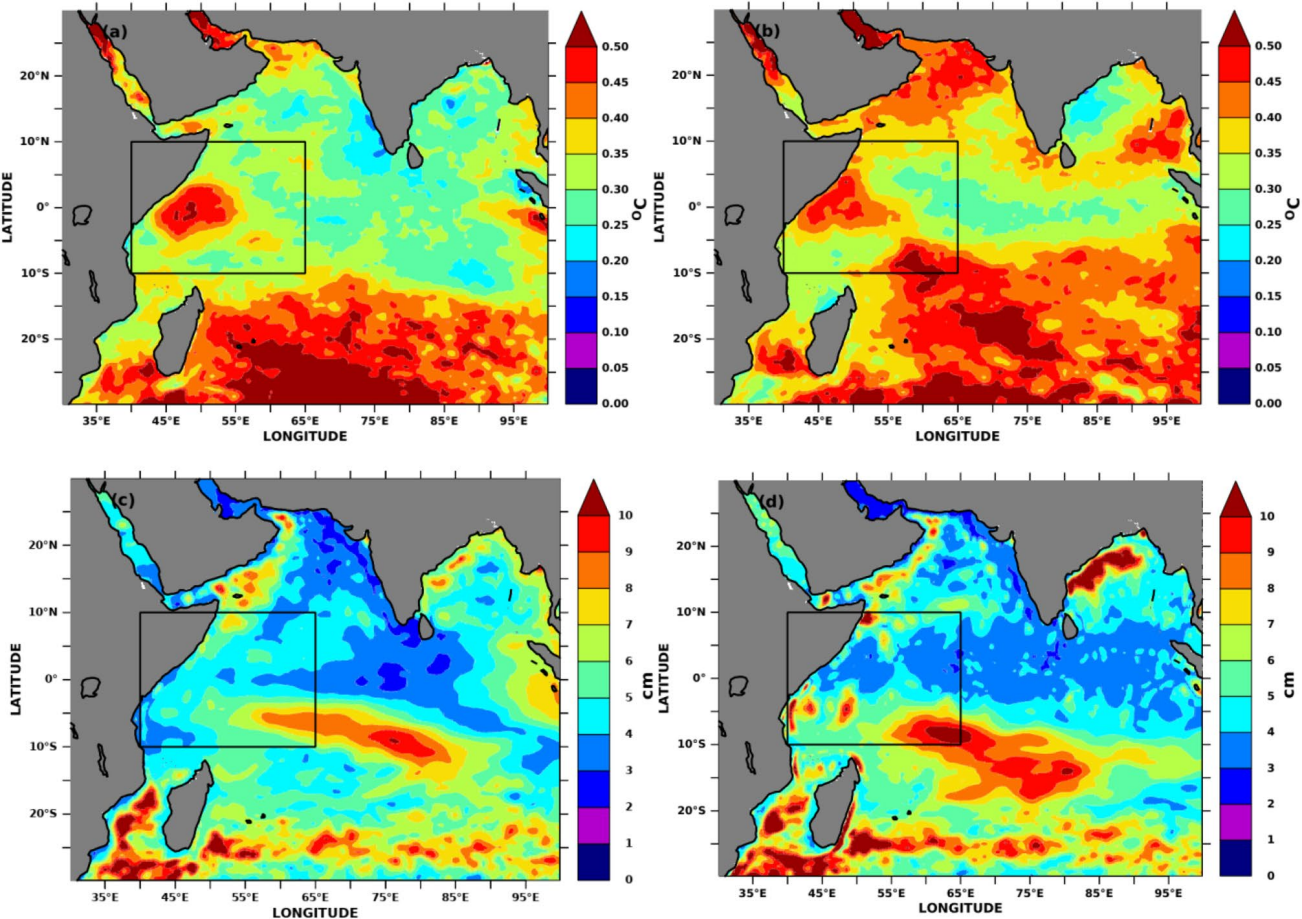
SSTA and MSLA standard deviation in the Indian Ocean. SSTA standard deviation during (**a**) winter months (Nov–Jan) (**b**) summer months (Mar–May). MSLA standard deviation during (**c**) winter months (Nov–Jan) and (**d**) summer months (Mar–May). Standard deviations are computed by using the annual means for 27 years. The figure is generated using PyFerret v7.63.

During the winter half-year, the southward movement of the intertropical convergence zone could lead to less insolation and ocean warming and change the SSTA and MSLA over SWIO. Therefore, these changes in insolation and air-sea fluxes coupled with altering wind patterns would induce differences in SSTA and MSLA along the SWIO and the total upper ocean heat capacitance. The increase in insolation due to northward propagation of the intertropical convergence zone and increased wind speeds due to land-sea pressure gradient will cause SSTA, MSLA and the total heat content available in the upper ocean to increase in summer months over SWIO. Nevertheless, SSTA standard deviation over SWIO is the highest in the Indian Ocean in the winter and summer months before the onset of summer monsoon over India (Fig. 4a,b). Furthermore, the large variability during Mar–May is associated with a robust cross-equatorial flow of winds over this region. This substantial variability on the positive side over the region favours the evaporation and the magnitude of precipitation received over land in the following months (June–September). MSLA, on the other hand, shows moderate standard deviation values over the SWIO but has a significant standard deviation over the Seychelles-Chagos thermocline ridge region^82^ propagating from the southeast during the winter months (Fig. 4c). The magnitude has become stronger during the summer months and will reach the SWIO (Fig. 4d), thus contribute to increasing the heat capacitance over the region.

### SSTA and MSLA relationship in the Indian Ocean

**Figure 5:**
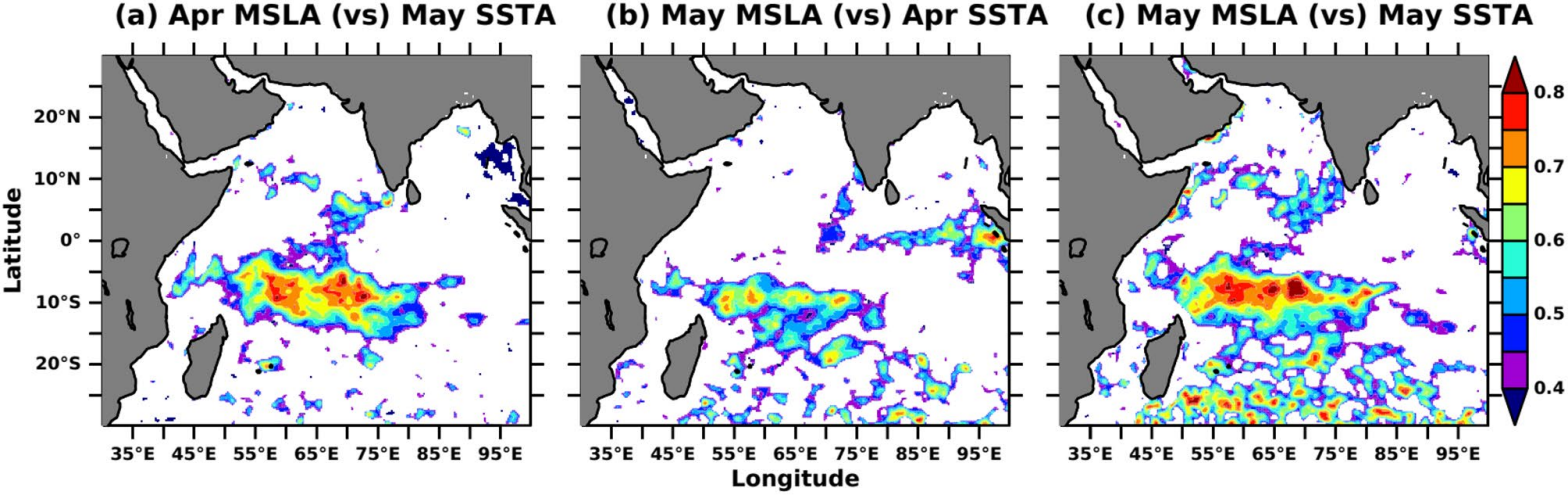
Lagged and simultaneous correlation of detrended SSTA and detrended MSLA during April–May in the study period. Areas with 95% significant correlations are shown. The figure is generated using PyFerret v7.63.

Xie et al.^83^ have shown that the thermocline variability over the SWIO affects the SST variability. This variability is due to the remotely controlled dynamical effects of phenomena like ENSO over the Pacific Ocean and IOD over the Indian Ocean^49^ and subsequent changes in the warming and heat over this region. The air-sea interaction process over this region dominates the SST variability on seasonal and intra-seasonal time scales and is mostly driven by upper ocean variability^82^. In order to see whether SSTA drives MSLA or MSLA drives SSTA, we use simultaneous and lead-lag spatial correlation. Figure 5 shows the spatial correlation pattern between SSTA and MSLA. Figure 5a shows the correlation between April MSLA and May SSTA. The correlation is high over the SWIO and maximum over the Seychelles-Changos thermocline ridge region. The spatial extent and correlation values over this region are reduced when the correlation between May MSLA and April SSTA is used (Fig. 5b). The aerial extent and correlation values are maximum for the simultaneous time i,e., for both MSLA and SSTA for the month of May. It is to be noted that simultaneous and lagged correlation patterns between MSLA and SSTA retain almost similar features, but if they reversed i,e., for May MSLA and April SSTA, the correlation values reduced drastically with reduced spatial extent. This suggests that the MSLA is determining SSTA through the change of thermocline depth over this region and the upper ocean heat capacitance. Also, correlation values are high over the eddy-rich southern Indian Ocean (Fig. 5c). This suggests the ocean eddy also affects SSTA.

### Impact of Indian Ocean SSTA on SAI

**Figure 6:**
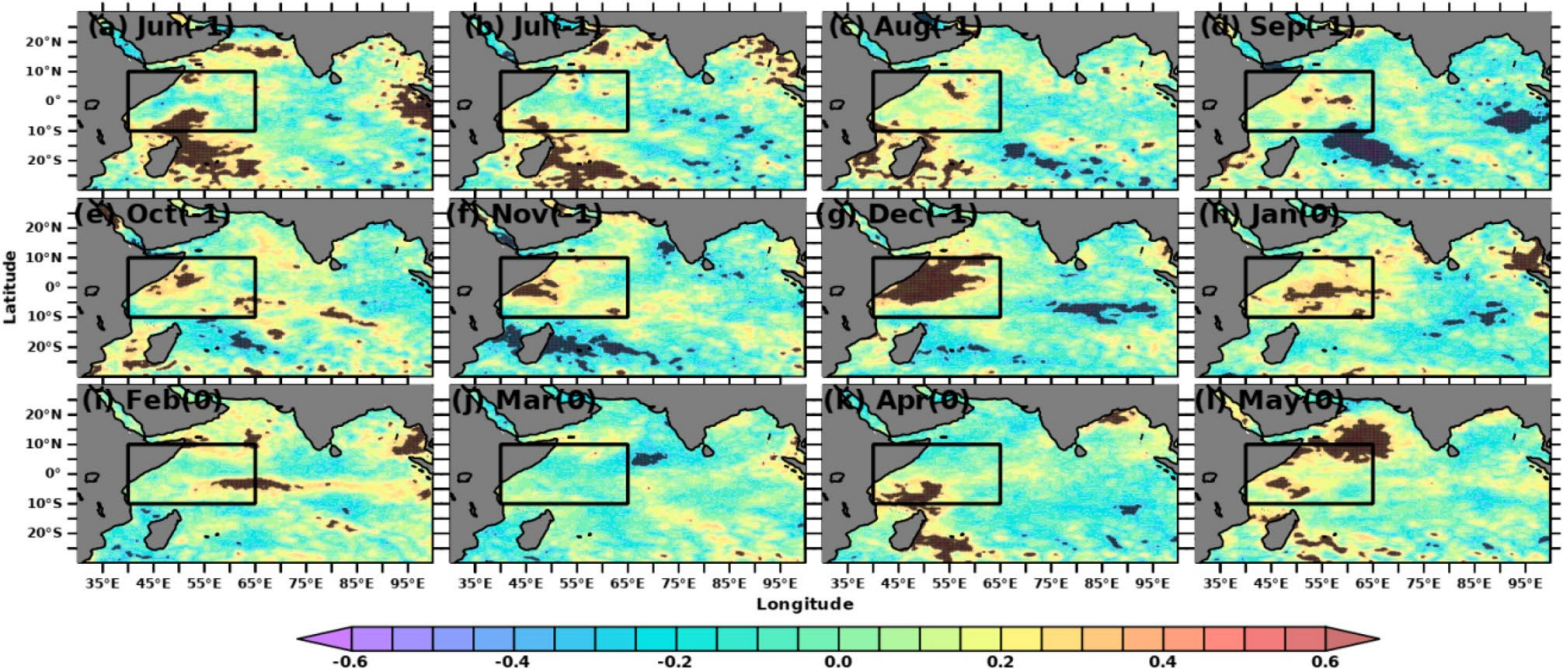
Lagged correlation of detrended AISMR SAI with detrended SSTA over the Indian Ocean. Areas with 90% significance are overlayed with black dots. The figure is generated using PyFerret v7.63.

The role of Indian Ocean SSTA in affecting the total AISMR has been discussed in several previous studies^84–87^. Here we correlated the AISMR SAI with the preceding year’s (− 1) June month to the current year’s (0) September month’s SSTA and MSLA over the Indian Ocean. Figure 6 shows the spatial lagged correlation with values significant at a 90% level between AISMR SAI and SSTA from June (− 1) to May (0). The Pearson’s correlations were computed between current year AISMR SAI and SSTA from the previous year’s June to the current year’s May. It shows that the current year’s SSTA over the central Arabian Sea and the eastern equatorial Indian Ocean, which is connected to the Indo-Pacific warm pool, can affect the following year’s monsoon. This may be because of persistent warm waters and ocean eddies life cycles over these regions^88–90^ affecting the upper ocean heat content over the region. The lagged correlation shows the maximum spatial extent over the SWIO during December–January. These results also corroborate the earlier findings of Kothawale et al.^32^. Thereafter, the spatial extent of significant correlation starts to decrease and diminished by the current year’s March. It reappeared again in April over SWIO and over the southwestern Arabian Sea during May, favouring the monsoon onset over the west coast of India. High correlations over the southwestern Arabian Sea correspond to strong upwelling caused by the winds during this time^33^. So, observing and understanding prevailing synoptic conditions over this region prior to monsoon onset (during early spring) could lead to better seasonal forecasting of the AISMR^91^. Also, this gives sufficient lead time for the seasonal prediction of AISMR together with homogeneous regions. On the other hand, the skill of AISMR seasonal prediction using CFSv2 is maximum using February initial conditions^92,93^.

**Figure 7:**
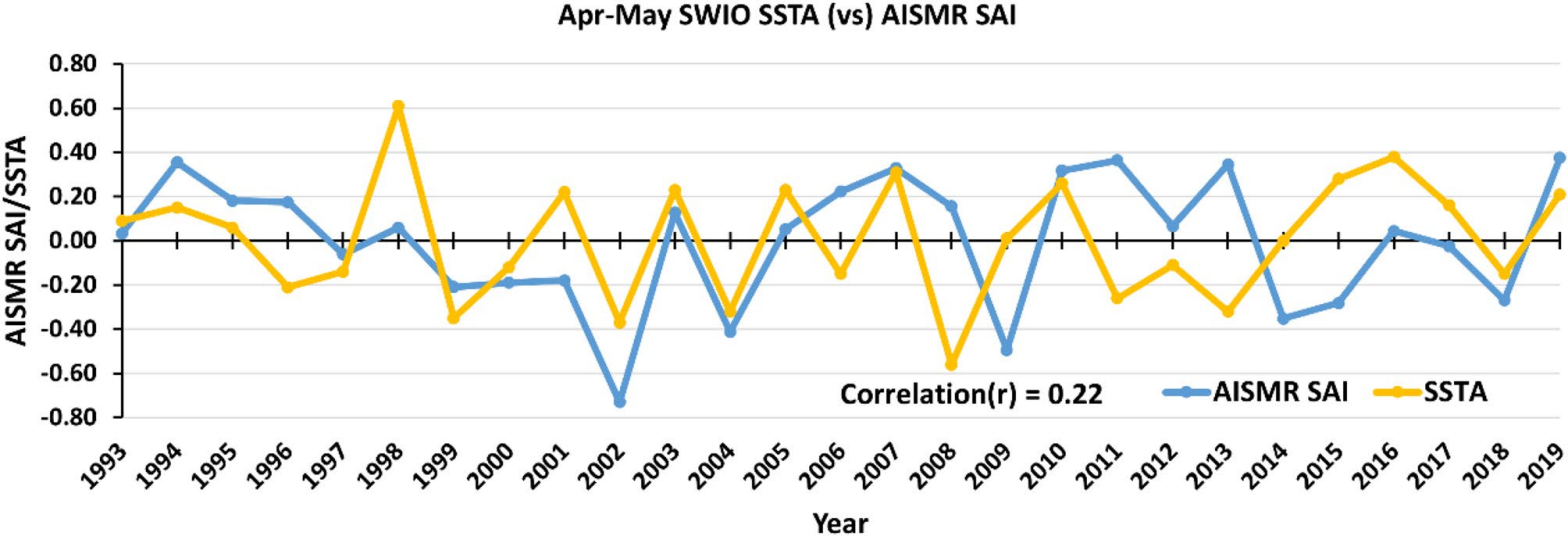
Time series of detrended Apr–May mean SSTA (°C, yellow) over SWIO compared with detrended AISMR SAI (blue). The figure is generated using Microsoft Excel.

In the previous section we find that April–May SSTA over SWIO may affect the SAI (Fig. 2 and 5). In order to see its inter-annual relationship, we show the time series of Apr-May SSTA over SWIO (40–65° E, 10° S–10° N) and AISMR SAI in Fig. 7. The Apr–May SSTA variability over SWIO shows a correlation of 0.22 with AISMR SAI. Among other atmosphere–ocean factors, the increasing trend in SSTA due to increasing total heat capacitance over the region in recent times could be one of the reasons favouring the large interannual variability of AISMR. These results differ slightly from the similar work of Vecchi and Harrison^94^ and Izumu et al.^33^ as their findings show AISMR being correlated to SSTA over the Arabian Sea and off Java and Sumatra. However, this result corroborates the finding of Simon et al.^95^. The robustness of the relationship can be attributed to evaporative moisture flux from this region induced by high wind stress^35^. Earlier studies show that the bulk of the water vapour flux comes from the 10–20 S zonal belts, including the SWIO region^96,97^.

**Figure 8:**
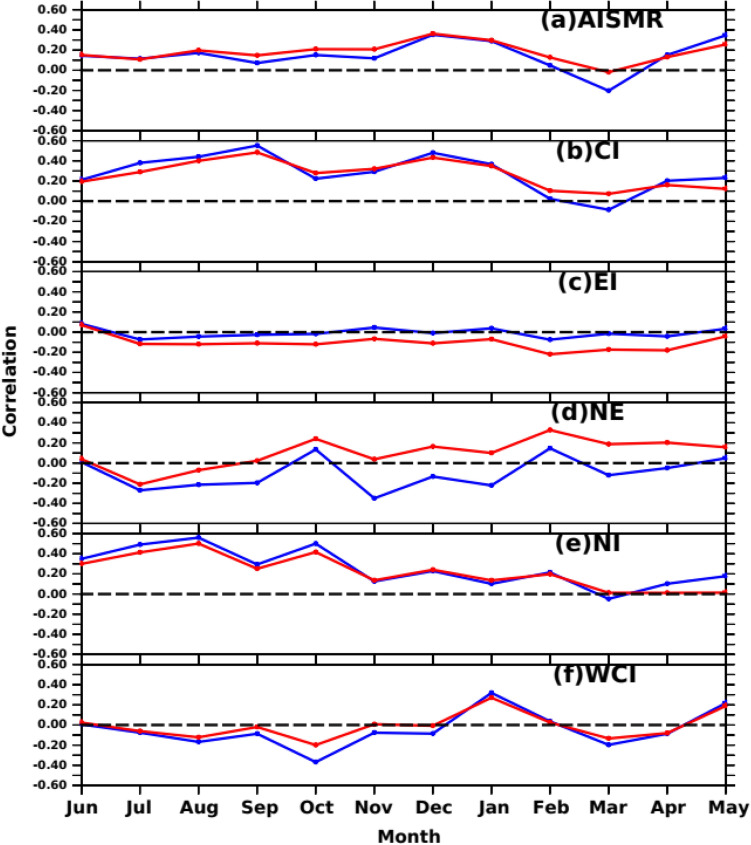
Time series of lagged correlation between detrended SSTA over SWIO and detrended regional rainfall (SAI) over the five homogeneous regions and entire Indian landmass before (red) and after (blue) removing ENSO effect. The figure is generated using PyFerret v7.63.

Venugopal et al.^35^ and Ali et al.^34^ showed that ocean mean temperature derived from ocean heat content/capacitance over SWIO could be a better oceanic predictor for AISMR. Nevertheless, the question remains: Do the SWIO SSTA and subsurface dynamics and variability (MSLA) affect the summer monsoon rainfall over the entire Indian landmass or only the homogeneous regions? To the best of our knowledge, no such studies have reported how the SWIO SSTA and MSLA will affect the different subregions of rainfall. On the other hand, a large part of the interannual variability of monsoon rainfall is linked to ENSO, a coupled ocean-atmospheric phenomenon in the Pacific Ocean. This phenomenon leads to the large-scale displacement of the east–west circulation in the tropics, influencing global SSTA^98,99^. Given this, the impact of ENSO on the relationship between Indian Ocean SSTA/MSLA and SAI (including the homogeneous regions) has been examined by removing the influence of the ENSO effect using Eq. 2, shown in Sect. 3.3. Figure 8 shows the time series of lagged correlation (Jun (− 1) to May (0)) between SSTA over SWIO and regional rainfall over the five homogeneous regions and the entire Indian landmass (AISMR) before (red) and after (blue) removing the ENSO effect. We consider the Nino3.4 region SSTA as the indicator of the ENSO phenomena. The ENSO effect has been removed following the Eq. 2, described in Sect. 3.3. This figure provides information on two aspects 1. How the antecedent month’s SSTA over SWIO impacts the rainfall over different sub-division i,e., homogeneous regions. 2. In which region ENSO effect is strongest? It can be seen that the correlation is maximum in Dec-Jan and April–May which was also reflected in the spatial correlation (Fig. 8a). It also shows the correlation values increase in a few months after removing the ENSO effect. It can also be seen that entire Indian mainland (AISMR) is affected by ENSO-induced SWIO SSTA such that correlations are lower after removing the ENSO effect. Out of five homogeneous regions, the monsoon rainfall over the CI and NI was positively affected by the antecedent month’s SSTA changes over SWIO (Fig. 8b,f). However, removing the ENSO effect over SWIO SSTA during April–May positively affects the NI rainfall. Similarly, CI correlations increase without the ENSO effect, and ENSO does not affect the WCI rainfall (Fig. 8f). The ENSO effect was most prominent in the NE and EI regions (Fig. 8c,d). Rainfall over the NE region is sensitive to the ENSO^100,101^, with significant differences in correlations with and without removing the ENSO effect. The rainfall over Gangetic plains (EI) and WCI is related to negative SSTA over the SWIO during Oct-Nov of the previous years^94^. On the other hand, these regions get most of their annual rainfall during the southwest monsoon season.

Hence, it can be concluded that the antecedent month’s SSTA anomaly over SWIO independently affects the AISMR subregions, especially over NI and CI. EI and NE show opposite trends in correlations, with the ENSO effect dominating NE.

**Figure 9:**
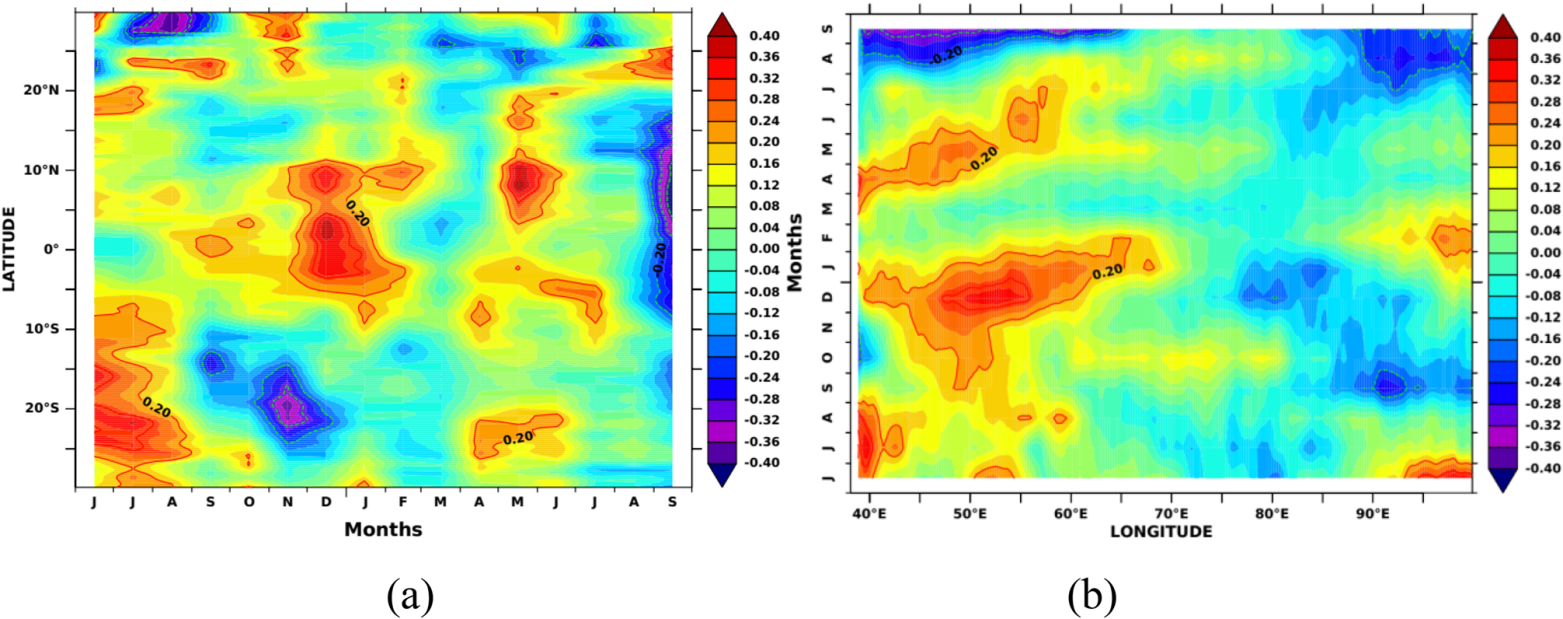
(**a**) Monthly lagged cross-correlation between detrended AISMR SAI and detrended SSTA averaged over 40-65E and (**b**) over 10S-10N. The figure is generated using PyFerret v7.63.

In order to see how the SSTA evaluation over SWIO affects the AISMR, we show the Hovemoller diagram of monthly lagged cross-correlation between AISMR SAI and SSTA averaged over SWIO (40–65 E) along 30 S–30 N for Jun (− 1) to September (0) in Fig. 9a. The large correlation values from 5 S to 10 N can be seen during Nov–Jan, which is also seen in Fig. 2. The correlations were confined to the equatorial region during pre-monsoon, i.e., from March and May. The decorrelation length between AISMR SAI and SSTA is ~ 3 months. Hovemoller diagram of monthly lagged cross-correlation between AISMR SAI and SSTA averaged over 10 S–10 N along 40 E–100 E is shown in Fig. 9b. It shows that the decorrelation length is localized over SWIO with a time length of ~ 3 months during pre-monsoon months. It propagates from the coast of Africa to till 60 E. The strong correlation between the previous year’s Oct to the current year’s Jan also shows longitudinal propagation from west to east. Both cases highlight the significant positive correlations over SWIO during the preceding autumn and winter months.

### Impact of Indian Ocean MSLA on SAI

**Figure 10:**
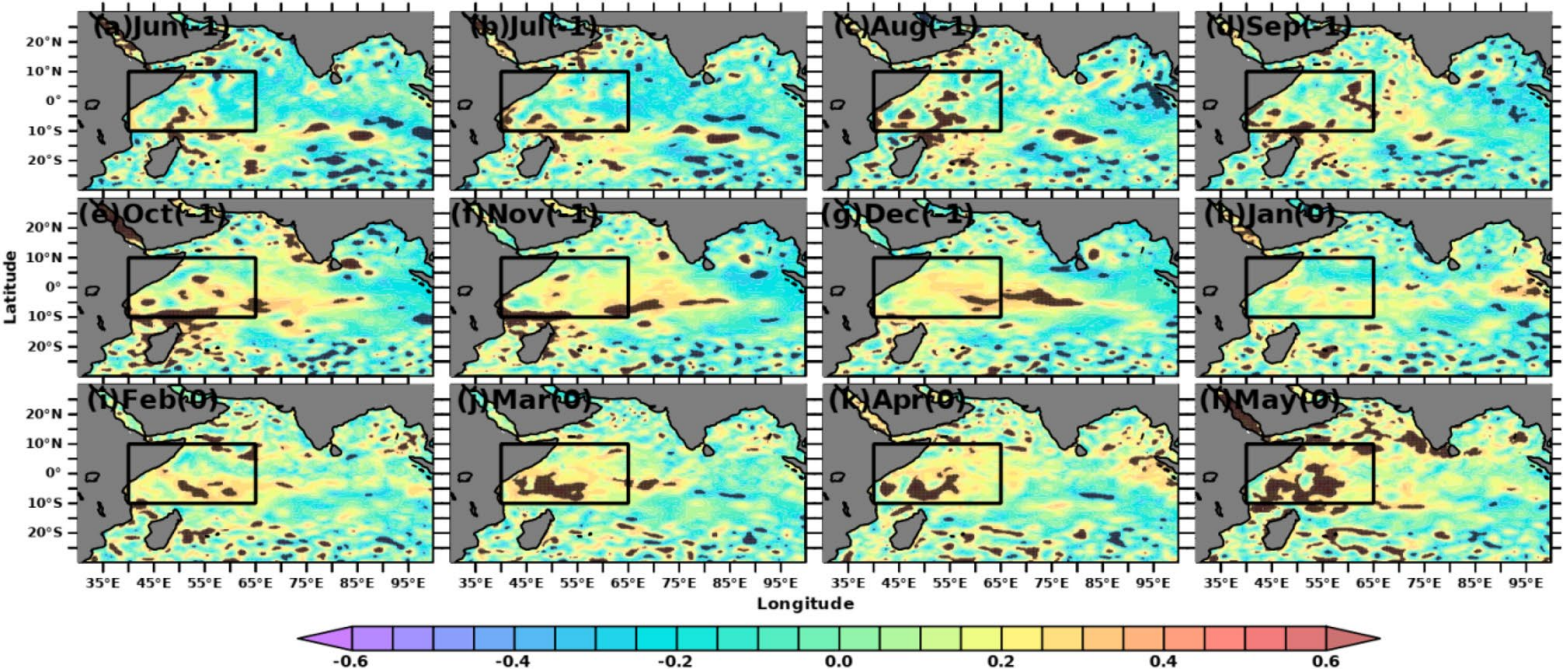
Lagged correlation of detrended AISMR SAI with detrended MSLA over the Indian Ocean. Areas overlapped with black dots are correlations with 90% significance. The figure is generated using PyFerret v7.63.

The correlation between MSLA from merged Altimeter data (MSLA) and AISMR SAI has been studied to explore the impact of subsurface variability on AISMR along with the surface air-sea interaction processes. MSLA variability gives the proxy of sub-surface temperature variability, which will further indicate the changes in the stored heat content in the ocean subsurface. Figure 10 shows the monthly lagged correlations between AISMR SAI and MSLA from June (− 1) to May (0) over the Indian Ocean. The correlation values follow a similar pattern as in the case of SSTA. Winter MSLA variability (Oct–Dec) over SWIO and along the western tropical Indian Ocean show a large influence on the following year’s AISMR. The correlation over SWIO has increased during the preceding October and continued until the current year’s May (0). While January (0) correlations are low, the spatial extent and correlation values slowly increase and tend to peak in the following months until May (0). As shown in Fig. 5, the correlation between MSLA and SSTA is maximum in the month of May, which shows SWIO MSLA affects AISMR. It is also interesting to note that significant correlation values are seen over mesoscale eddy locations. This shows the possible local influence of ocean eddy on large-scale convective activities. Thus, the sub-surface heat capacitance dominates during the pre-monsoon time, as in the case of SSTA. The correlation values above 90% significant levels observed over SWIO suggest that air-sea interactions over the surface, subsurface ocean dynamics and thermodynamics over the region play a key role in the AISMR variability.

**Figure 11:**
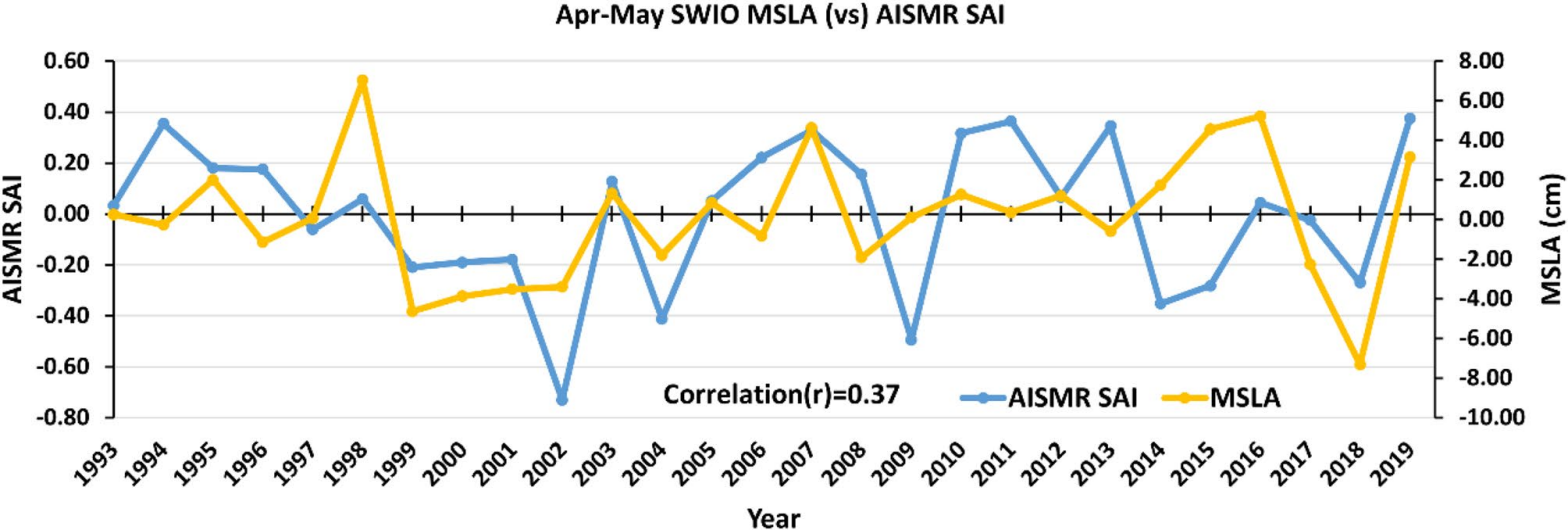
Time series of detrended Apr–May mean MSLA (yellow) over SWIO compared with detrended AISMR SAI (blue). The figure is generated using Microsoft Excel.

To see the effect of April–May MSLA on AISMR SAI on an inter-annual time scale, we show the time series of MSLA over SWIO and AISMR SAI in Fig. 11. A good match between MSLA and AISMR SAI was found with a correlation value of 0.37. This analysis shows MSLA potentially influences the SSTA over the region through air–sea flux exchanges possibly controlled by oceanic mixing process^102–105^. Mechanisms about how these physical and dynamic processes are influencing the AISMR will be studied in detail in our future studies.

**Figure 12:**
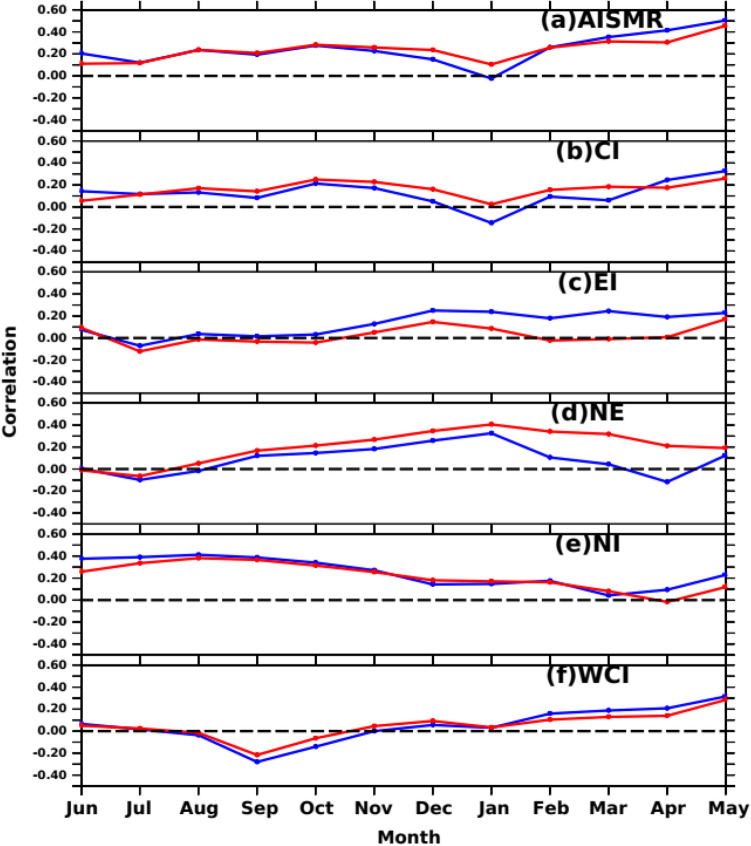
Time series of lagged correlation between detrended MSLA over SWIO and detrended regional rainfall (SAI) over the five homogeneous regions and entire Indian landmass before (red) and after (blue) removing ENSO effect. The figure is generated using PyFerret v7.63.

Like SSTA influence on AISMR SAI and other homogeneous regions, we also show MSLA influence on homogeneous regions. Figure 12 shows the time series of correlations between SSHA variations over SWIO and SAI. This relationship became robust during recent decades. This may be because Indian Ocean climate variability has become more active during the last couple of decades due to many positive Indian Ocean Dipoles^80,106,107^. Like SSTA, the lagged correlation time series between SAI and MSLA over SWIO shows robust MSLA effects and heat capacitance on the regional rainfall (Fig. 12). SAI and SSHA show positive peak correlations in the preceding December (− 1) and during the current year’s May (0). Except for NE and NI, rainfall over all other homogeneous regions shows no large coupling with the antecedent month’s MSLA variability over SWIO. The correlation of EI and NE rainfall is strongly impacted by ENSO-induced SWIO MSLA (Fig. 12c,d). On the other hand, correlations over other regions remain unchanged after removing the ENSO effect (Fig. 12), particularly during winter and pre-monsoon months. Thus, correlations over the NE region are sensitive to both MSLA and SSTA over SWIO. NE rainfall is caused by the Bay of Bengal branch of AISMR. Thus, the Bay of Bengal branch of the AISMR might have a strong coupling with the antecedent month’s SSTA and MSLA over SWIO. However, the impact of MSLA variability during pre-monsoon months on WCI and NE (Fig. 12b,f) rainfall could influence the AISMR interannual variability due to the large share of the rainfall over the two regions in the total magnitude of AISMR.

**Figure 13:**
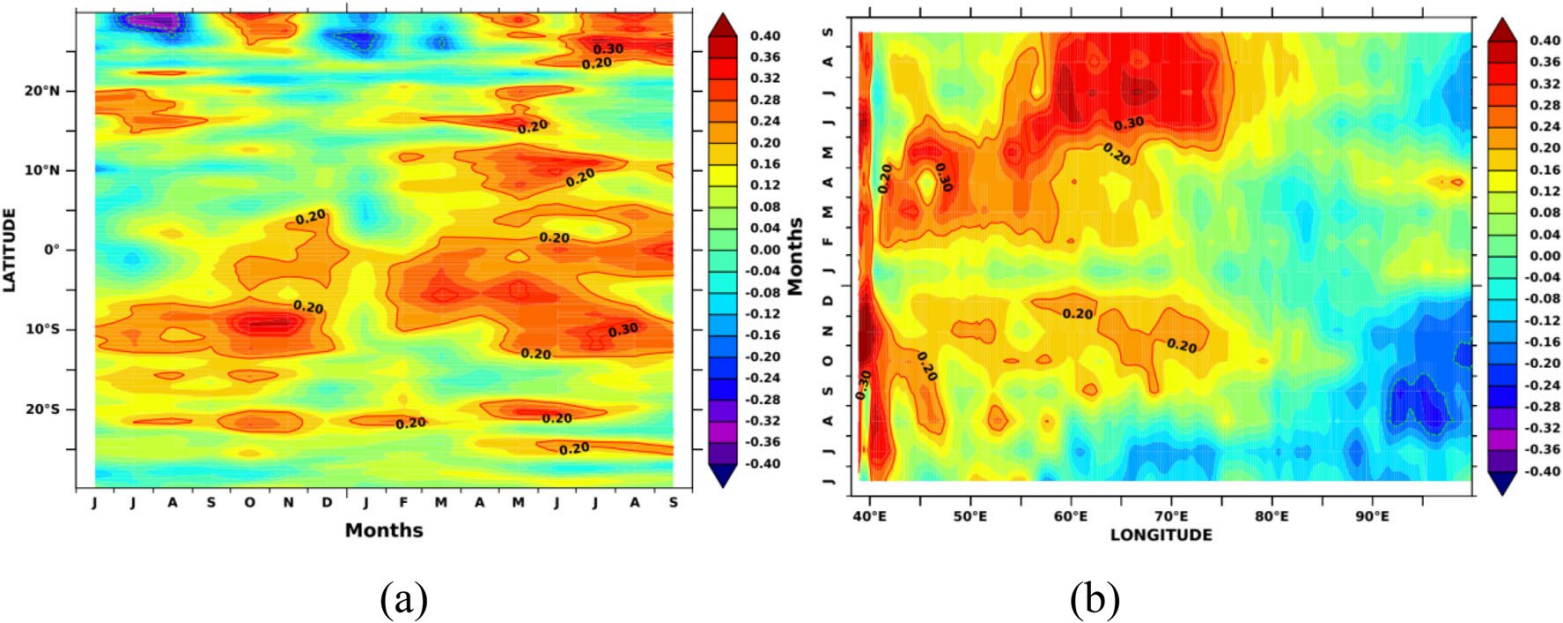
(**a**) Monthly lagged cross-correlation between AISMR SAI and MSLA averaged over 40-65E and (**b**) over 10S-10N. The figure is generated using PyFerret v7.63.

Figure 13a shows Hovemoller plots (like Fig. 9a) of lagged cross-correlations between AISMR SAI and MSLA averaged over 40–65 E along 30 S–30 N. The results show two maxima, one around the previous year’s Oct–Nov (− 1) and the other around Feb–Mar (0), lasting until Aug-Sep (0). The decorrelation scale is persistent over 15–20 S. However, the average over 10 S–10 N (Fig. 13b) along 40 E–100 E (like Fig. 9b) shows the decorrelation scale to propagate eastward during pre-monsoon months until the end of the same monsoon season (Fig. 13b). Thus, the MSLA variations due to subsurface ocean heat content over 40–65 E and 10 S–10 N have a significant role in the AISMR variability. The observed high simultaneous correlation values between AISMR SAI and MSLA during summer monsoon time support the hypothesis of Shankar and Shetye^37^. Accordingly, the seasonal inflow of the monsoon rainfall into the seas around India and the dynamics of currents along the Indian coast provide the link between the rainfall over the Indian subcontinent and the MSLA along the coast of India, with coastal salinity playing an intermediate role.

### Indian Ocean SSTA and MSLA variability during contrasting monsoon

**Figure 14:**
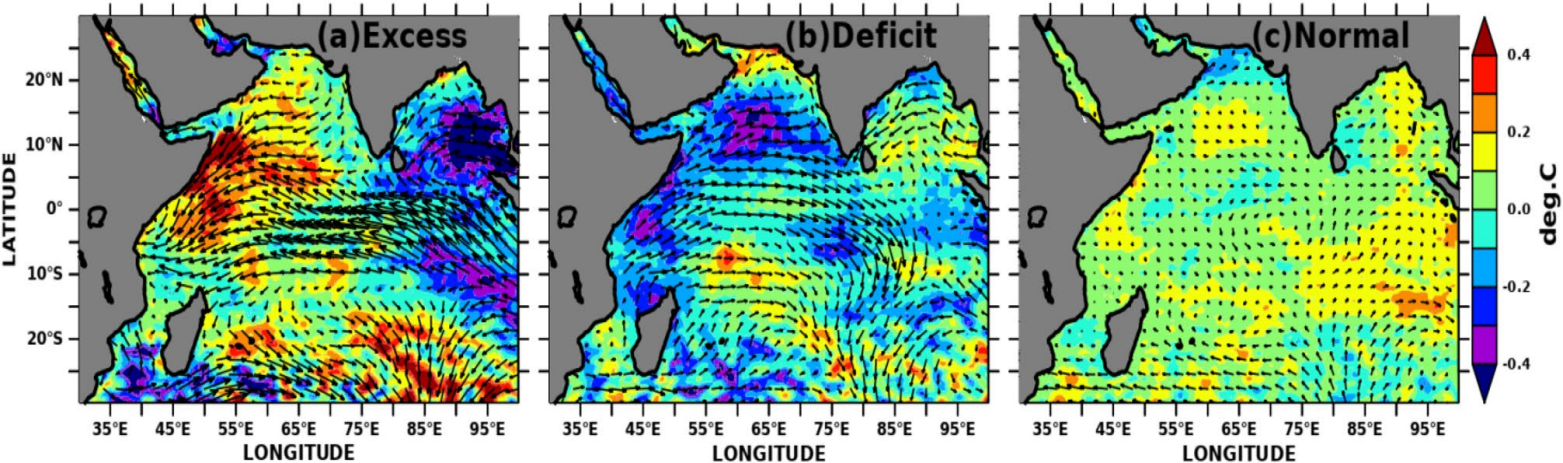
Composite of May month’s detrended SSTA variability (shaded) overlayed with detrended ERA5 wind anomalies (vectors) over the Indian Ocean during (**a**) excess, (**b**) deficit, and (**c**) normal monsoon years. The figure is generated using PyFerret v7.63.

To delineate how the SWIO impacts the extreme monsoon rainfall variability we have defined the excess, normal and drought years according to Mooley and Parthasarathy^72^. Figure 14 shows the composite of SSTA (shaded) and sea surface wind (vector overlayed) for excess, deficit, and normal monsoon years. During excess monsoon years (Fig. 14a), SSTA shows positive anomalies over the SWIO, including the Seychelles-Chagos thermocline ridge region. Similarly, anomalous weak surface winds (near the east African coast) with a north-easterly flow instead of normal south westerlies were observed. This anomalous weak wind reduces the upwelling and, thus, the cooling and produces conducive conditions for positive SSTA over this region, and the region becomes one of the moisture source regions during the subsequent monsoon months^33^. On the other hand, during the deficit years (Fig. 14b), the anomalous stronger south-westerly wind cools the SWIO and hence reduces the moisture source for the subsequent months^33^.

**Figure 15:**
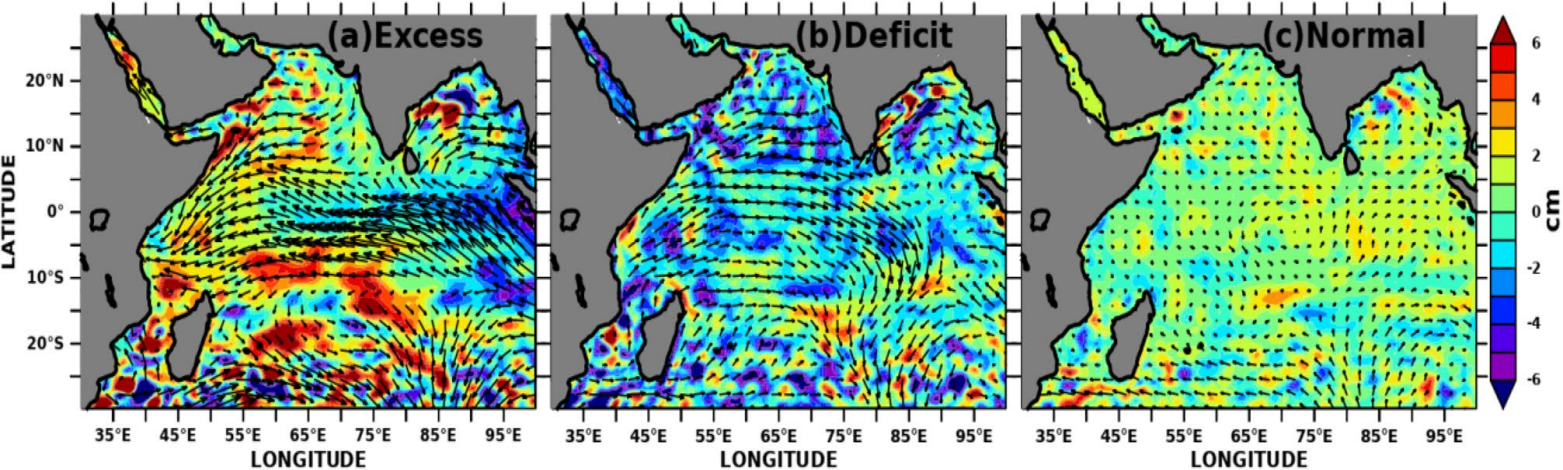
Composite of May month’s detrended MSLA variability (shaded) overlayed with detrended ERA5 wind anomalies (vectors) over the Indian Ocean during (**a**) excess, (**b**) deficit, and (**c**) normal monsoon years. The figure is generated using PyFerret v7.63.

The MSLA variability can be taken as a proxy for ocean heat content/capacitance. We show similar composite plots with MSLA in Fig. 15. The positive MSLA during excess monsoon years over the entire SWIO leads to deeper isothermal layers and could act as a heat source for the moisture supply (Fig. 15a). In contrast, the composite during the deficit monsoon years shows an opposite spatial pattern of MSLA and reversal of winds over the entire Indian Ocean (Fig. 15b). Both SSTA and MSLA over SWIO, including the Seychelles-Chagos thermocline ridge region, turned unfavorable with strong westerly winds. The negative MSLA signifies the deep mixed layer depth and shallow thermocline over these regions. Normal monsoon years are accompanied by moderate values of SSTA, MSLA and surface winds (Figs. 14c, 15c), which could lead to dampened air-sea flux exchange between the upper ocean and the lower troposphere over the Indian Ocean. The wind anomalies from National Centers for Environmental Prediction (NCEP) daily reanalysis 2 data with 2.5 × 2.5 resolution^108^ also show similar results (not shown here).

## Climatic effect of the Indian Ocean on AISMR

It has been shown in many previous studies that Indian Ocean SST is increasing due to global warming scenarios over the last several decades^109–112^ and a two-fold increase of the areal extent of the Indo-Pacific warm pool in recent time^113^. Due to this effect, the extreme monsoon rainfall events show an increasing trend, and moderate to low rainfall events show decreasing trend^114–116^. However, these studies also reported no long-term secular trend of AISMR from observed data but showed multi-decadal variation^71^. Despite no long-term secular change, few studies show a decreasing trend of AISMR rainfall over CI. One possible mechanism explaining this decline in CI rainfall is the westward shift of monsoon trough by 2–3° longitudes in association with warming of western Indian Ocean^117^. All these studies focused on the SST impacts on simultaneous time i,e., during JJAS. In this study, we show that the antecedent month’s SSTA and MSLA over SWIO, particularly during April–May (0), impacts AISMR and other homogeneous regions’ rainfall. Furthermore, we also show that SWIO mostly affects NE, NI and CI. In order to see the long-term climate change of AISMR with respect to Apr-May SST, we show the trend analysis in this section.

**Figure 16:**
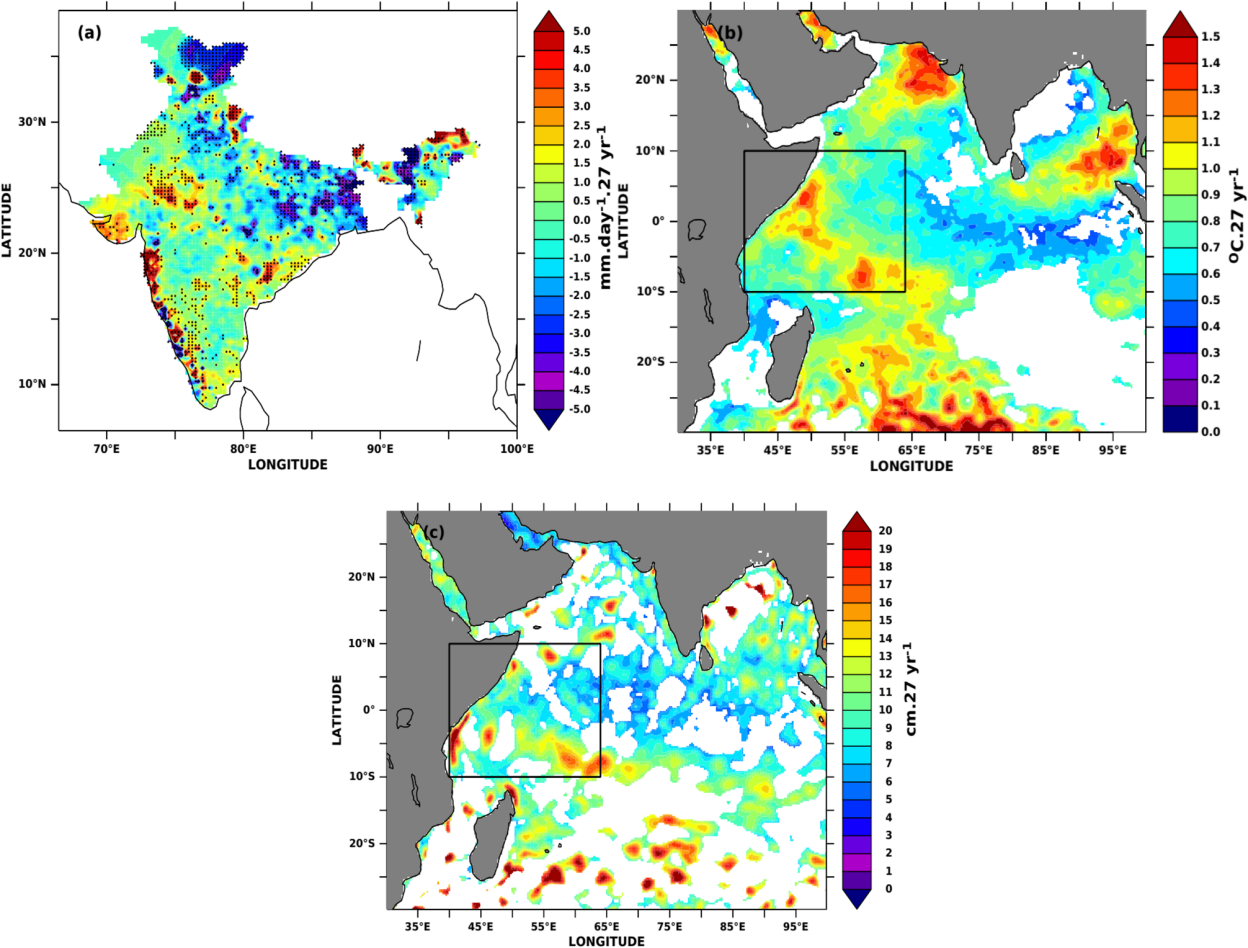
The linear trend for (**a**) AISMR, 90% significance trend is marked with black dots, (**b**) Apr–May SST, and (**c**) Apr–May SLA during 1993–2019. For both SST and SLA, areas with trends significant at 95% are plotted. The figure is generated using PyFerret v7.63.

Figure 16 shows the changes (linear trend) in the AISMR rainfall, sea surface temperature (SST), and SLA during 1993–2019. Except for peninsular India and western India, most of the regions show a decreasing trend. The decreasing trend is most prominent over EI and CI. This result corroborates the finding of Roxy et al.^114^, which shows AISMR decreasing trend over CI during 1901–2012 using IMD and climate research unit (CRU) observations. The study finds that the decreasing AISMR over CI is mainly due to the land-sea contrast of temperature because of Indian Ocean warming (Fig. 16a). In a recent study using the same Pai et al.^64^ data, Yadav and Roxy^118^ showed that in the recent two decades (1996–2017), northern India (78.5° E–87.5° E, 24° N–28.5° N) shows a decreasing trend with increased variability, much larger than the earlier period (1979–2000). The study finds that the rise in warm sea surface temperature (SST) observed in the eastern equatorial Indian ocean is possibly the reason for this decreasing rainfall trend over NI. Changing synoptic conditions and remote forcing, together with local climate change, have increased the frequency of extreme rainfall, flash floods, glacier collapse, and avalanches in the Himalayan regions in recent decades^119–121^. The extremes in rainfall over these regions further cause landslides, damage to property and infrastructure and loss of lives.

Figure 16b shows the SST trend for the months of April–May during 1993–2019 (27 years). It shows a significant annual trend over the northeast Arabian Sea, Southern BoB, western Indian Ocean, and southern Indian Ocean (~ 1–1.2 °C in 27 years). It shows negative trends over the eastern equatorial Indian Ocean. SLA also shows a similar spatial trend pattern to SST. It shows a positive trend of 7–10 cm in 27 years over the southwestern Indian Ocean, including the Seychelles-Tangos thermocline dome and southern Indian Ocean (Fig. 16c). These increasing trends are mostly seen in the mesoscale eddy structures. The long-term rise in SLA over these regions implies that the thermocline became deeper, leading to an increase in SST. Thus, the total upper ocean heat content/capacity is increasing over this region^36,122^. SWIO region also shows the most considerable interannual and intra-annual variability of upper ocean parameters among other parts of the global tropical oceans due to its connection with coupled phenomena such as summer monsoon, IOD, ENSO^49,123–126^. Both SSTA and MSLA along the monsoonal low-level jet path in the SWIO show a substantial change from summer half-year (April–September) to winter half-year (October–March)^36,127,128^. Thus, a slight change in the magnitude of these parameters over SWIO during antecedent months could lead to changes in the upper ocean heat capacitance which will further tend to significant changes in air-sea interactions from the ocean surface to the lower troposphere, cloud cover, variability in radiation, and turbulent fluxes over this region. Though the difference in half-year wind speeds is moderate over the region, the atmosphere is saturated with water vapour, and the influx of continuous moisture from the ocean into the atmosphere would lead to a surplus monsoon rain season^129–131^.

**Figure 17:**
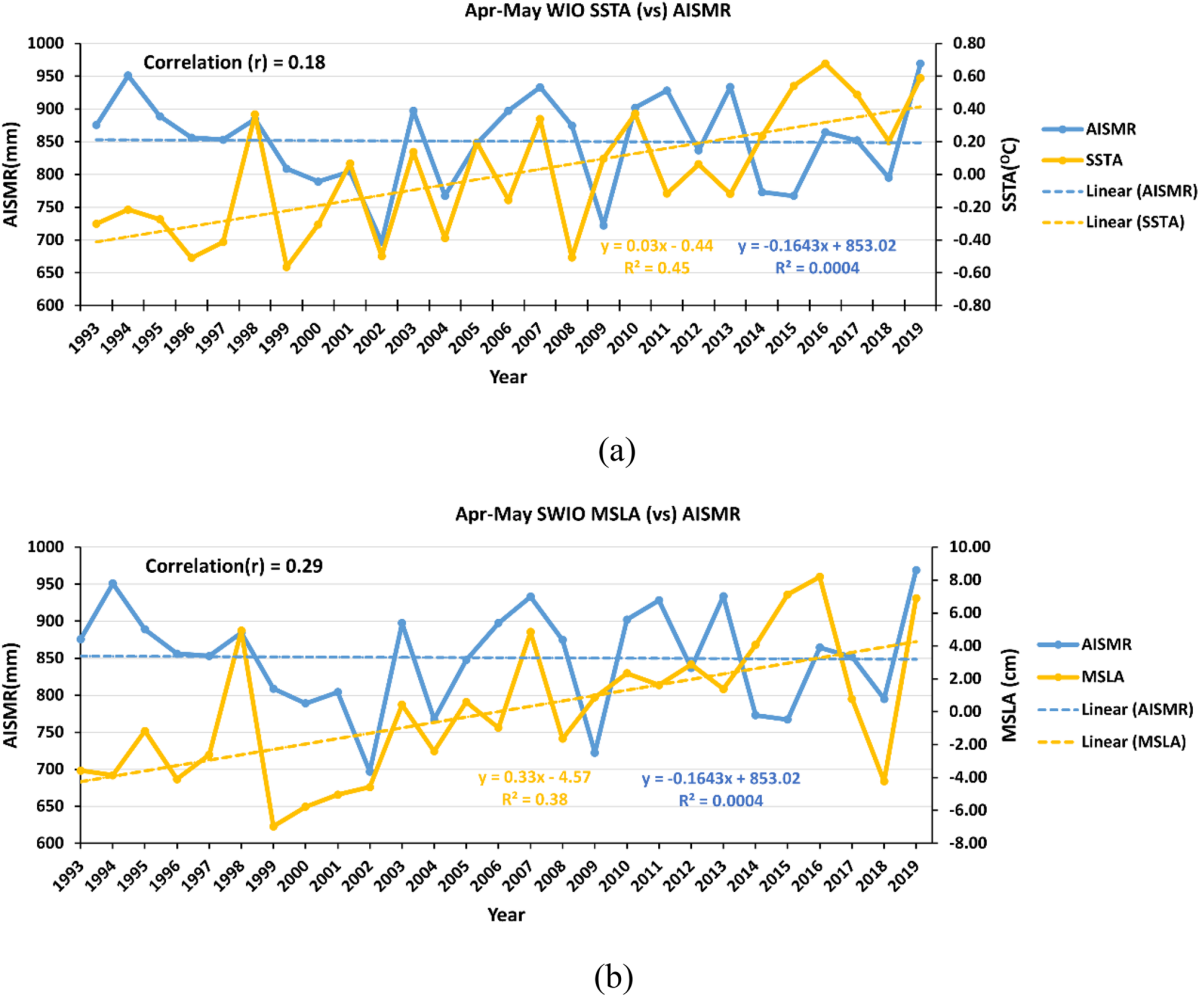
Time series of AISMR and SWIO (**a**) Apr–May SSTA, and (**b**) Apr–May MSLA during 1993–2019. The figure is generated using Microsoft Excel.

Figure 17 shows the April–May inter-annual time series of SSTA and MSLA with AISMR over the SWIO. The increasing trend in SSTA and MSLA over SWIO does not show any long-term change in AISMR, with correlations of 0.18 and 0.29, respectively. However, rainfall over NI, NE and EI has shown decreasing trend in the recent decade (2006–2019) with increasing Apr-May SWIO SSTA and MSLA trends (Figures S3, S4). Rainfall over CI and WCI show no such trend during the study period. Thus, the recent decade (2010–2019) witnessed the highest number of excess monsoon years (3) and the lowest number (4) of normal monsoon years, and only three (3) years being deficit monsoon years.

## Summary and conclusions

The availability of high-resolution satellite altimeter (SLA) and microwave SST data opens a window into a detailed study of the Indian Ocean’s impact on AISMR for the last three decades. Several recent studies have highlighted the effects of pre-monsoon SST and upwelling in the Arabian Sea and the tropical Indian Ocean^132–134^. In addition, several recent studies have investigated the role of subsurface variations in the form of ocean heat content, mean sea surface temperature and sea level on AISMR^34,35^. However, most of these studies show the Indian Ocean effect on a simultaneous time scale. In this study, we focused on the pre-monsoon signal of the Indian Ocean and how it affects AISMR's spatial distribution, unlike the previous studies, which focused on AISMR as a whole. Several past studies attempted to understand the impact of the Indian Ocean on the variability of AISMR. These works have either focused on the total variability of AISMR or considered only single or multiple ocean parameters to study the contemporary relationship with AISMR. Thus, limited attempts were made to understand the connection between rainfall variability over homogeneous regions and oceanic parameters, such as heat capacitance in the Indian Ocean influenced by events like ENSO that occurred during the preceding months of AISMR. This study tried to address this aspect using long-term satellite-derived SST and SLA over the Indian Ocean.

The oceans, due to their large heat capacity, may influence the climate modes such as IOD and ENSO and subsequently affects the AISMR. Over the Indian Ocean, SWIO is a very dynamic region where air-sea interaction plays a prominent role in SST and SLA variability and, subsequently, AISMR^34–36,50^. Lead-lag correlation between MSLA and SSTA shows strong coupling over SWIO during April–May, and MSLA affects SSTA (Fig. 5). Thus, the strong coupling of MSLA and SSTA via SWIO resulted in strong seasonal variation of these two parameters across regions. Biannual changes in insolation due to wind reversal, changes in upper ocean parameters, and movement of the thermal equator make the SWIO region one of the world’s highest winter-to-summer SSTA and MSLA variability. Thus, it has become unique among other regions in recent time. Therefore, these two parameters show significant standard deviations over SWIO. Lead-lag correlations of Nino3.4 SSTA with both SSTA and MSLA over the Indian Ocean show significant correlation values during the previous year’s winter and the current year’s spring (Figs. 2 and 3).

SWIO heat capacitance in the form of SSTA and MSLA variability during preceding winter and pre-monsoon months induce inter-annual variability of AISMR over Indian landmass. SSTA over SWIO from the prior year’s September (− 1) to the current year’s February (0) month shows significant highest correlations over the SWIO with AISMR SAI (Fig. 6). The correlations have become more prominent over the Arabian Sea during May (0). We found the spring SSTA (April–May) over SWIO affects the inter-annual AISMR variability. However, SWIO impacts limits to the WCI, NI and CI rainfall, the rest of the land mass does not affect by the SWIO SSTA variability. This association even becomes stronger with the removal of ENSO, which suggests SWIO even independently impacts WCI, NI and CI rainfall variability. The ENSO effect becomes prominent through SWIO over the NE and EI rainfall variability. It is worth mentioning that studies show feedback of the Indian Ocean on ENSO weekend since the early 1990s ^135^. Similar impacts like SSTA are also found with MSLA. Hovemoller diagram for AISMR SAI correlations with SSTA, MSLA over SWIO shows a decorrelation length of ~ 3 months during winter and spring. The composite SSTA and MSLA spatial distribution for April–May shows large positive (negative) SST and MSLA anomaly over SWIO and the western Indian Ocean during excess (drought) monsoon years. This suggests prolonged heat source over SWIO is controlling the WCI, NI and CI rainfall variability through the supply of moisture during the monsoon season.

In recent times, many studies showed increasing Indian Ocean SST since the many decades. Its effect on AISMR is still debatable and not very conclusive. The long-term Apr–May SSTA and MSLA change did not show any effect on the long-term secular change of AISMR (Fig. 17). However, it may hold anonymous signal on the increase in extreme events as well as the decrease of moderate to low rainfall events. To the best of our knowledge, none of the previous studies show how the long-term change of pre-monsoon (April–May) SSTA and MSLA affects homogeneous region’s rainfall. In this study, we show the similar spatial distribution of AISMR long-term change during 1993–2019, which was reported in previous studies^114,115^. Despite increasing April–May SSTA and MSLA trends over SWIO and the western Indian Ocean, AISMR does not show any long-term secular change. However, the effect of long-term pre-monsoonal SSTA and MSLA shows decreasing rainfall trend over NI, NE, and EI in the recent time. No such trends were observed over WCI and CI, thus making the rainfall excess or normal in the recent decade. The advective heat transport due to Antarctic warming^136^, and Indo-Pacific warm waters^137^ may be causing warming and positive trends in SSTA and MSLA across SWIO. Also, possibly contemporary changes in Indian Ocean SSTA and MSLA causing large interannual variations in AISMR magnitude, resulting in more frequent droughts and floods in recent decades, as evidenced by the presence of 3 droughts in the 2010–2019 decade as compared to previous decades with 4 (2000–2009) and no draughts during 1993–1999. Finally, as the AISMR is a coupled atmosphere–ocean phenomenon, the ENSO influence on the atmosphere be dominating the ocean part in modulating the inter-annual variability of the AISMR.

## Supplementary Information

Supplementary Information.

## Data Availability

All data used in the study are freely available. Rainfall data can be obtained from India Meteorological Department (https://www.imdpune.gov.in/Clim_Pred_LRF_New/Grided_Data_Download.html). Sea surface temperature data was downloaded from National Oceanic and Atmospheric Administration (https://www.ncei.noaa.gov/products/optimum-interpolation-sst). Sea level anomaly data are obtained from Copernicus Marine Environment Monitoring Service (https://marine.copernicus.eu). Royal Netherlands Meteorological Institute hosts the NINO3.4 indices (https://climexp.knmi.nl/selectindex.cgi). ERA5 surface wind data was downloaded from the C3S climate data store (CDS) (https://cds.climate.copernicus.eu/cdsapp#!/dataset/reanalysis-era5-single-levels?tab=overview), and NCEP reanalysis from National Centers for Environmental Prediction (https://psl.noaa.gov/data/gridded/data.ncep.reanalysis.pressure.html).

## Code availability

The analysis was done using PyFerret developed by Pacific Marine Environment Laboratory, National Oceanic and Atmospheric Administration (https://ferret.pmel.noaa.gov/Ferret). The analysis codes used in this study are available from the corresponding author upon request.
