# Supplementary material for: Quantifying the role of antecedent Southwestern Indian Ocean capacitance on the summer monsoon rainfall variability over homogeneous regions of India
Source: Sci Rep. 2023 Apr 5;13:5553. doi: 10.1038/s41598-023-32840-w (PMC10076287; doi:10.1038/s41598-023-32840-w)
Supplement: Supplementary file 1 — Supplementary Information. [file 41598_2023_32840_MOESM1_ESM.docx]

**Supplementary Material**

**Quantifying the role of antecedent Southwestern Indian Ocean capacitance on the summer monsoon rainfall variability over homogeneous regions of India**

Venugopal Thandlam^*1,2,3,6^, Hasibur Rahaman^4^, Anna Rutgersson^1,3^, Erik Sahlee^1^, M Ravichandran^5^ and S.S.V.S Ramakrishna^6^

1. Air, Water and Landscape Science (Luval), Department of Earth Sciences, Uppsala University, Uppsala, Sweden.
2. The Center for Environment and Development Studies Research Forum, Uppsala University, Uppsala, Sweden.
3. Centre of Natural Hazards and Disaster Science, Uppsala University, Uppsala, Sweden.
4. Indian National Centre for Ocean Information Services, Ministry of Earth Sciences, Ministry of Earth Sciences, Hyderabad, India.
5. Earth System Science Organization, Ministry of Earth Sciences, New Delhi, India.
6. Department of Meteorology and Oceanography, Andhra University, Visakhapatnam, India.

*Corresponding author’s email: [venu.thandlam@geo.uu.se](mailto:venu.thandlam@geo.uu.se)


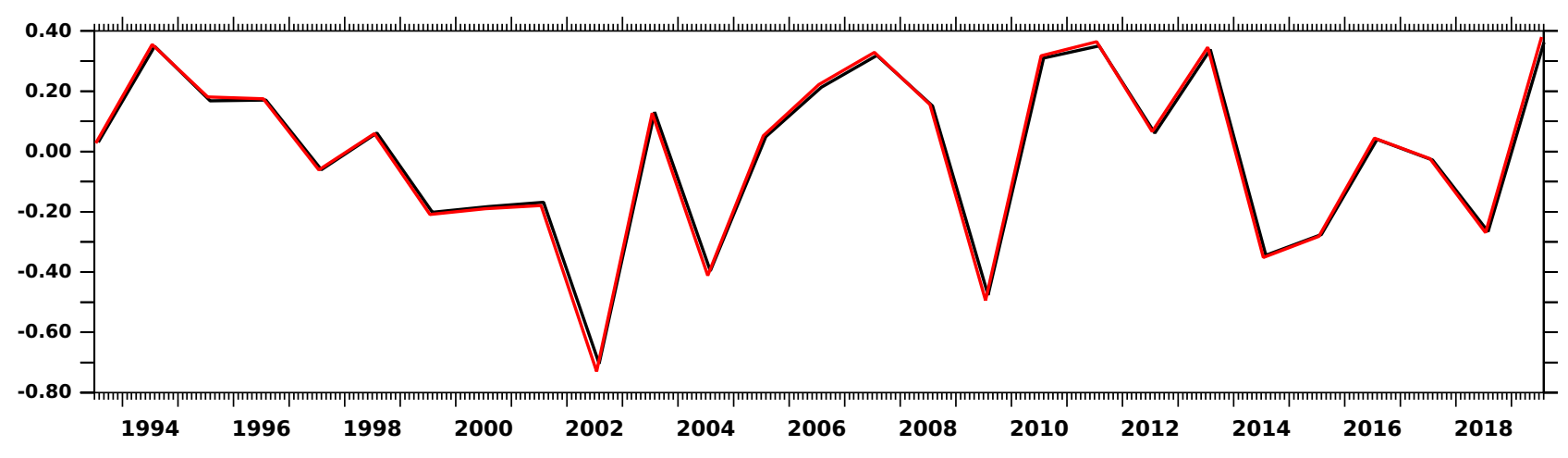


(a)


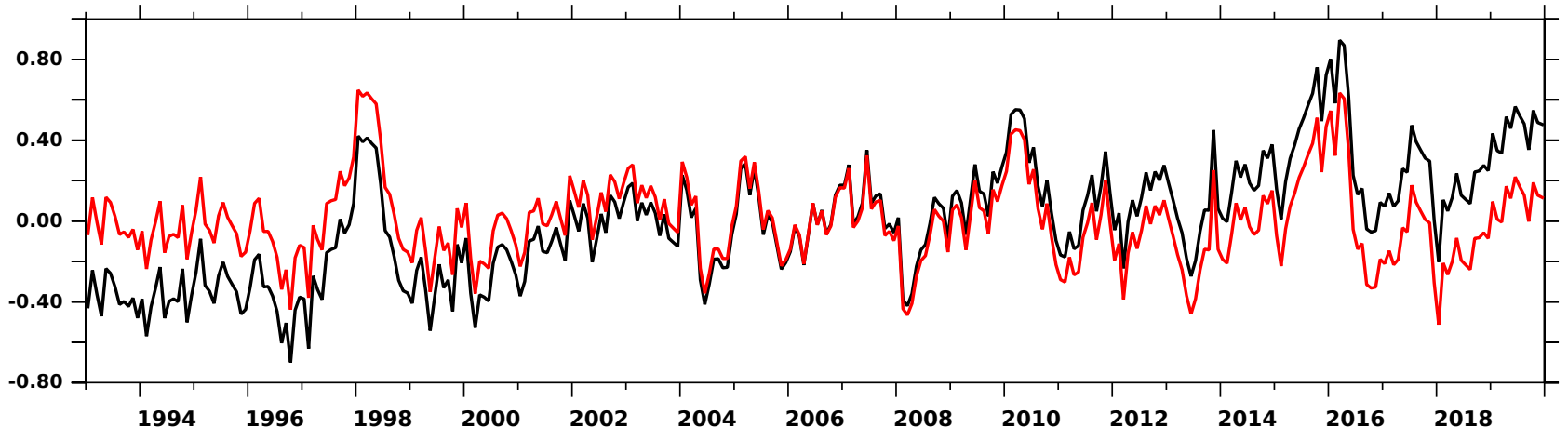


(b)


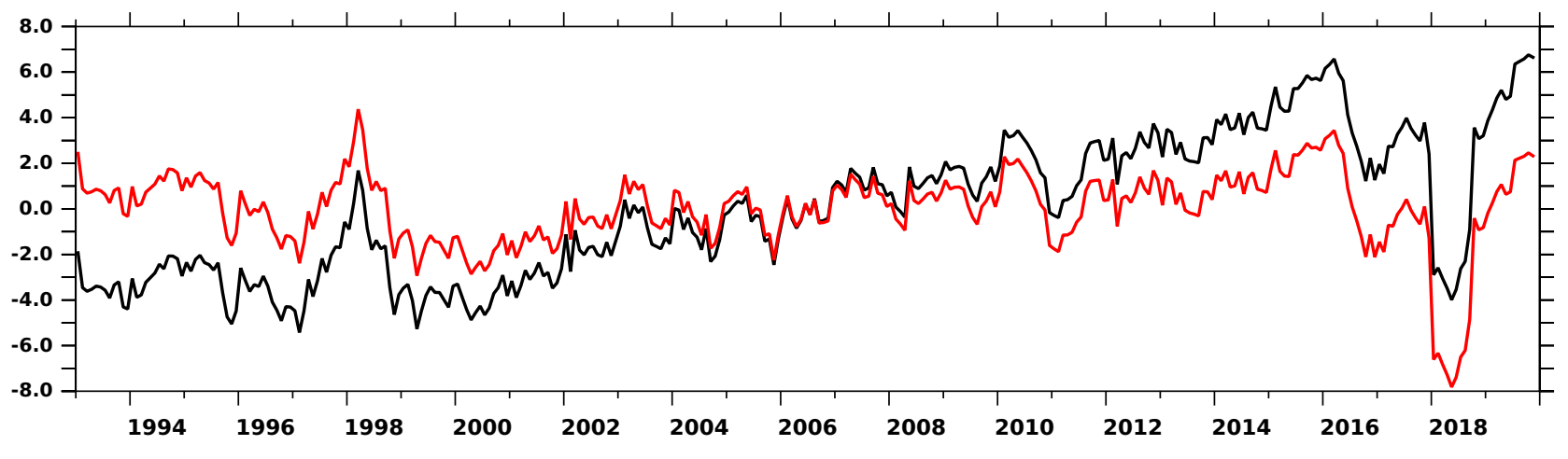


(c)


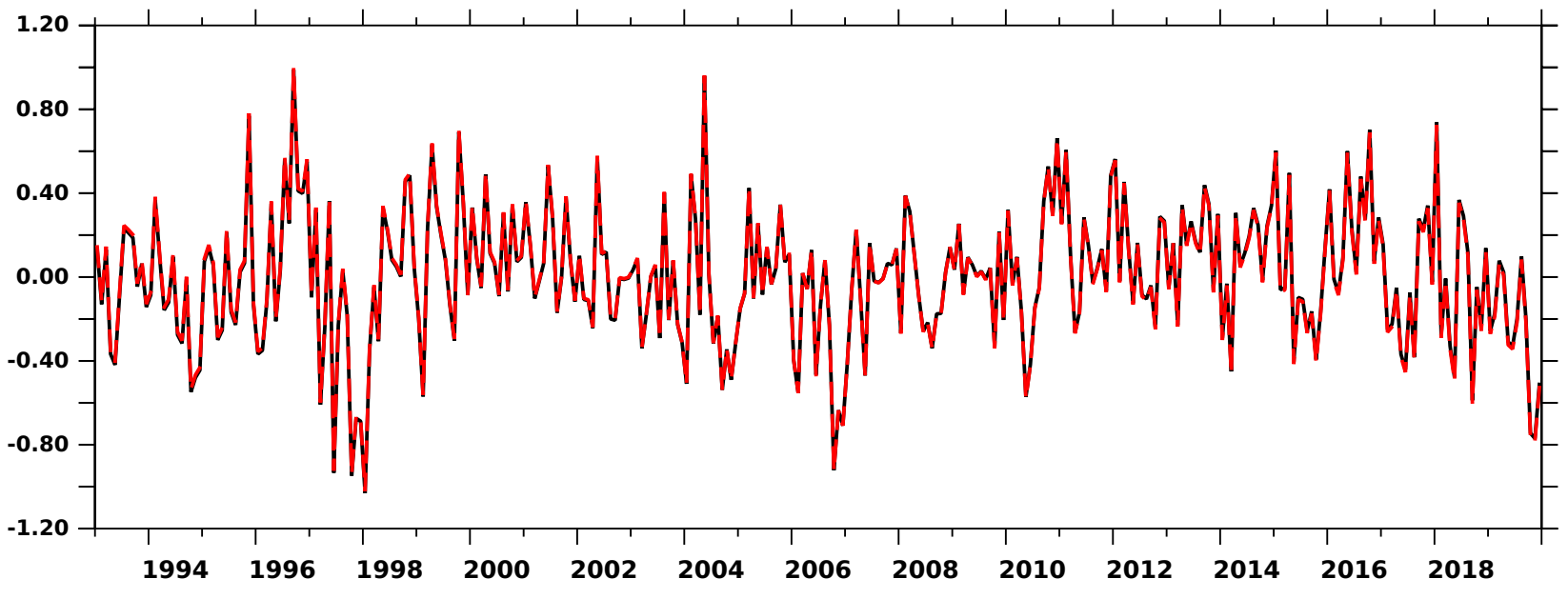


(d)


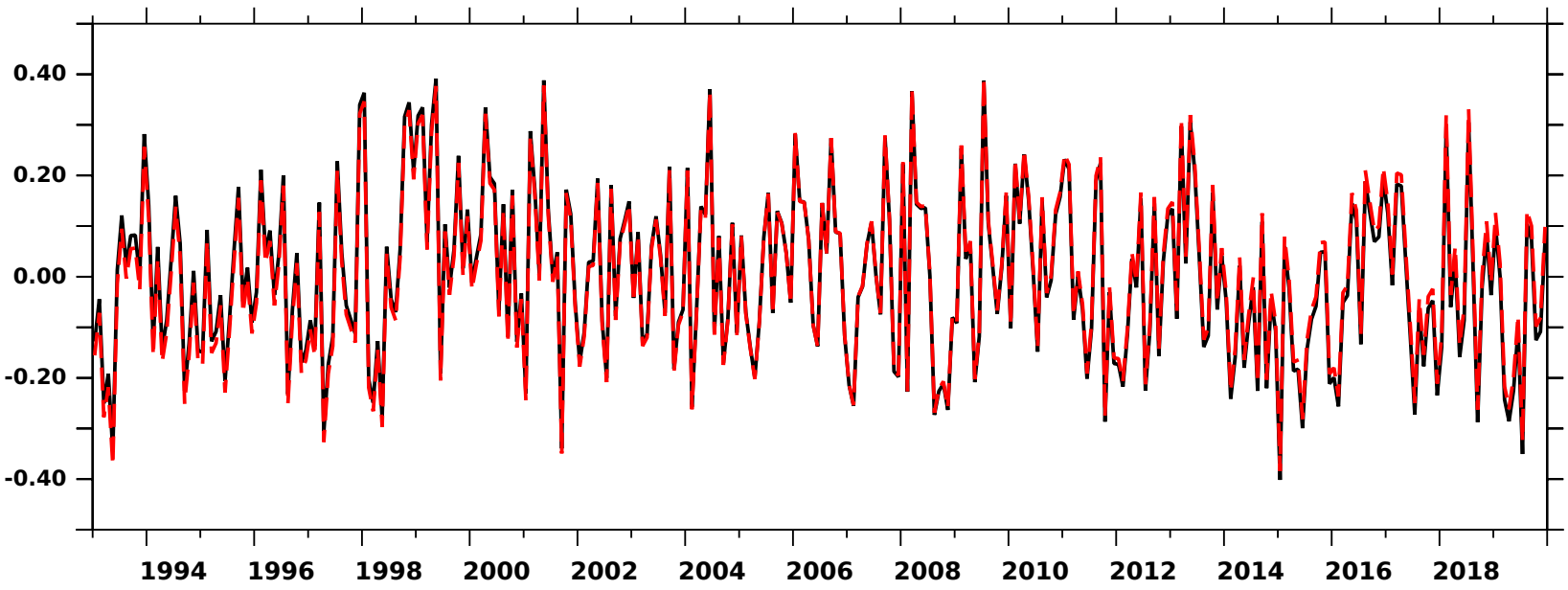


(e)

Figure S1: Time series of original (red) and detrended (black) data for (a) AISMR SAI, Indian Ocean monthly (b) SSTA (^o^C) (c) MSLA (cm) (d) zonal wind (m) and (e) meridional wind (m) during the study period. The figures are generated using PyFerret v7.63.


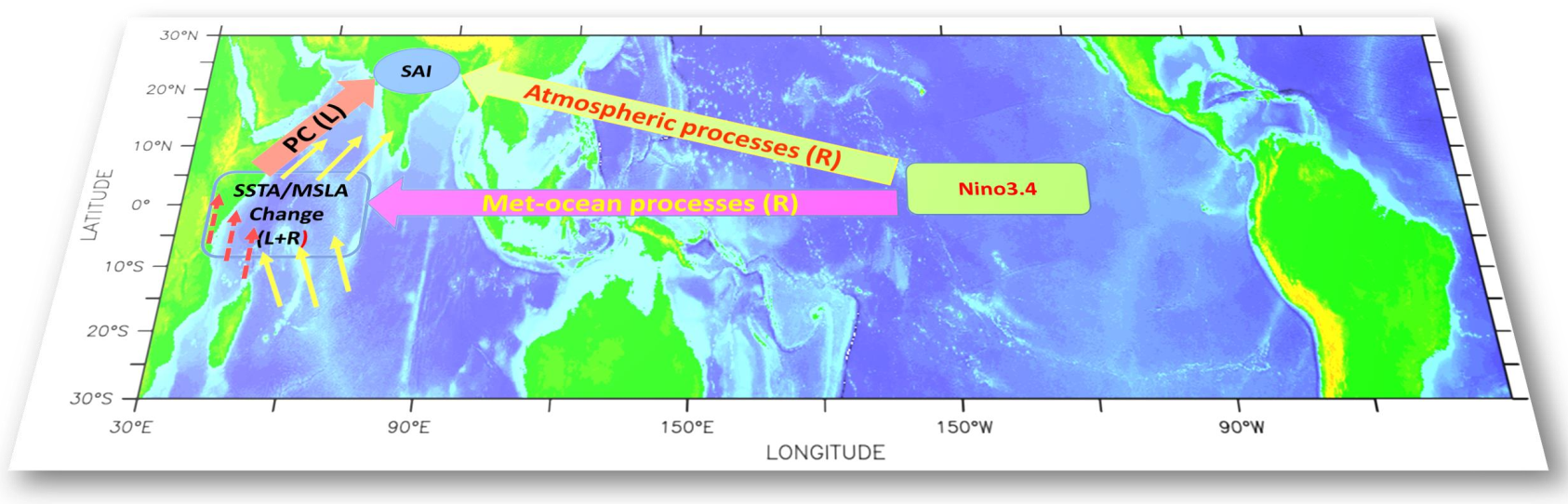


Figure S2: Schematic showing the current work. SAI: JJAS rainfall standard anomaly index over India. Nino3.4 represents the state of the ENSO. R: Changes in SAI over India and antecedent SSTA/MSLA over SWIO due to remote forcing induced by Nino3.4. L: local effects changing the antecedent SSTA/MSLA over SWIO leading to altering winds (yellow arrows) and evaporation (dashed red arrows) and thus effecting the rainfall (SAI). PC(L): Pearson’s correlations between SAI and antecedent SSTA/MSLA over SWIO after removing the Nino3.4 effect (remote forcing, R) on SAI and SSTA/MSLA. Schematic is generated using Microsoft Powerpoint and PyFerret v7.63.


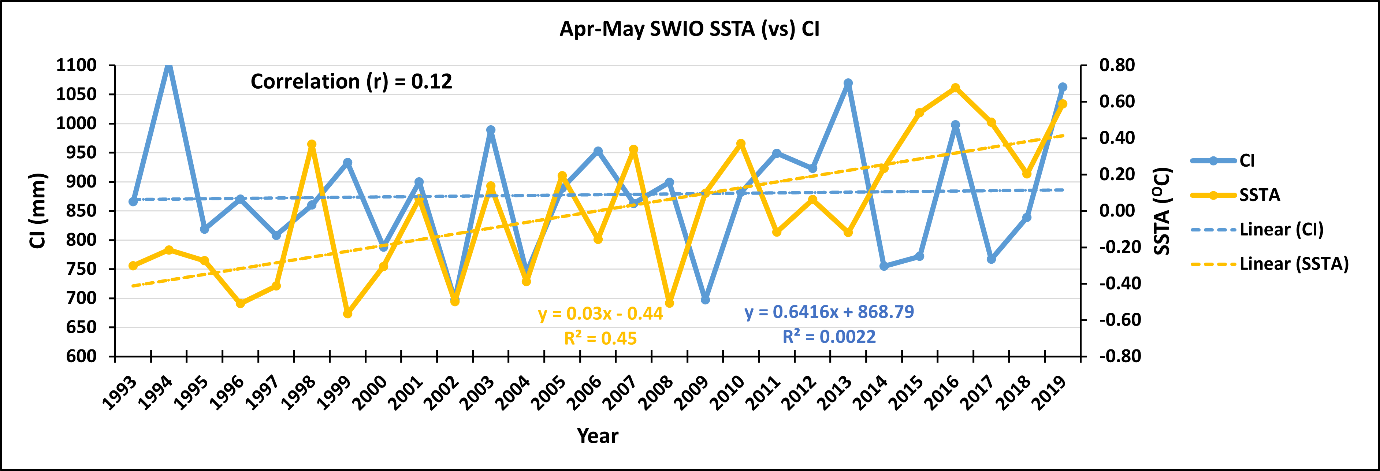


(a)


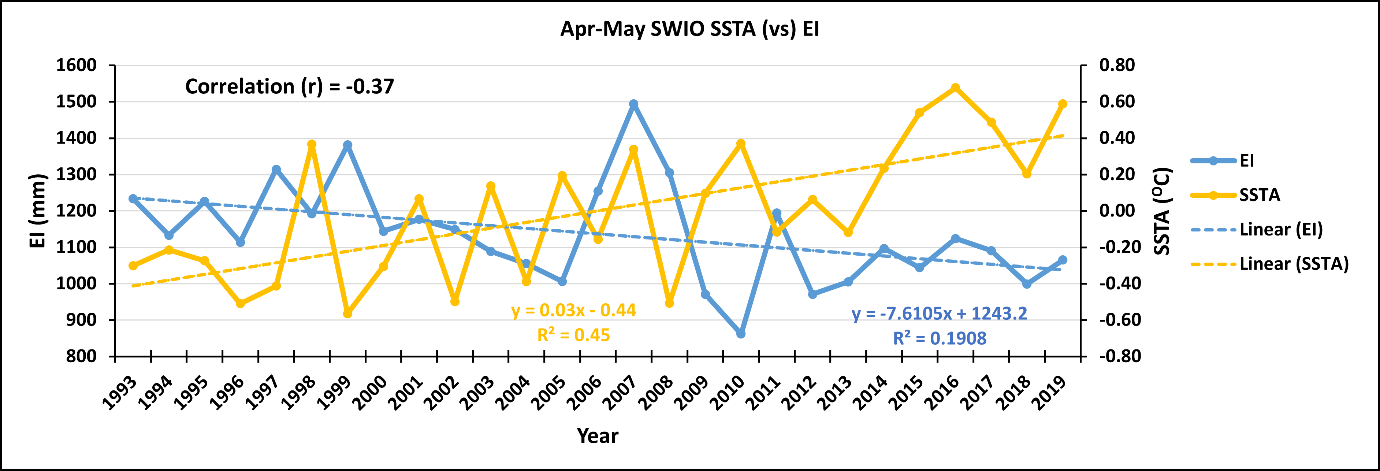


(b)


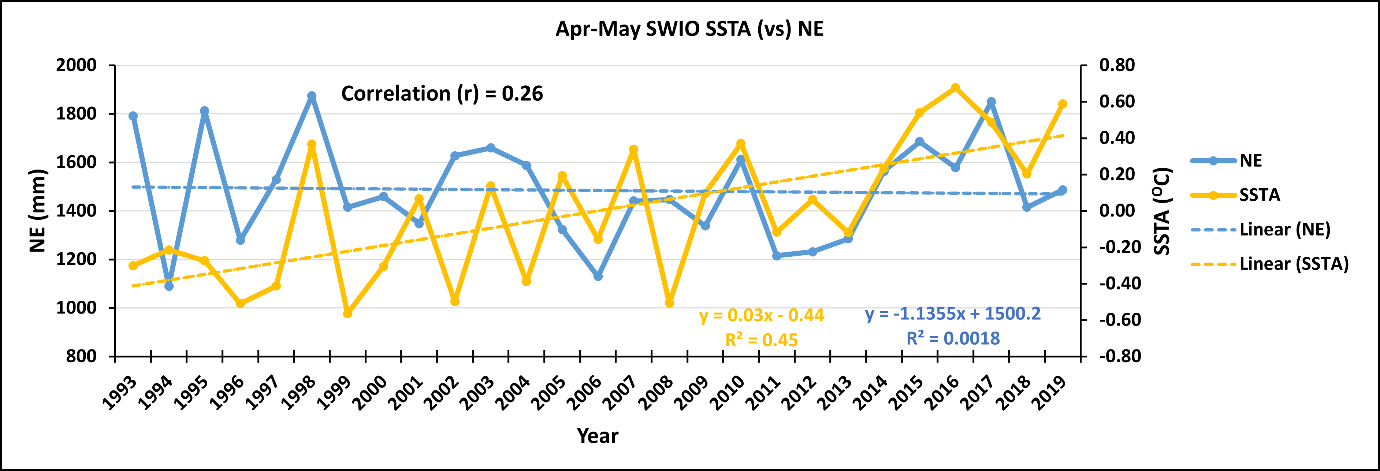


(c)


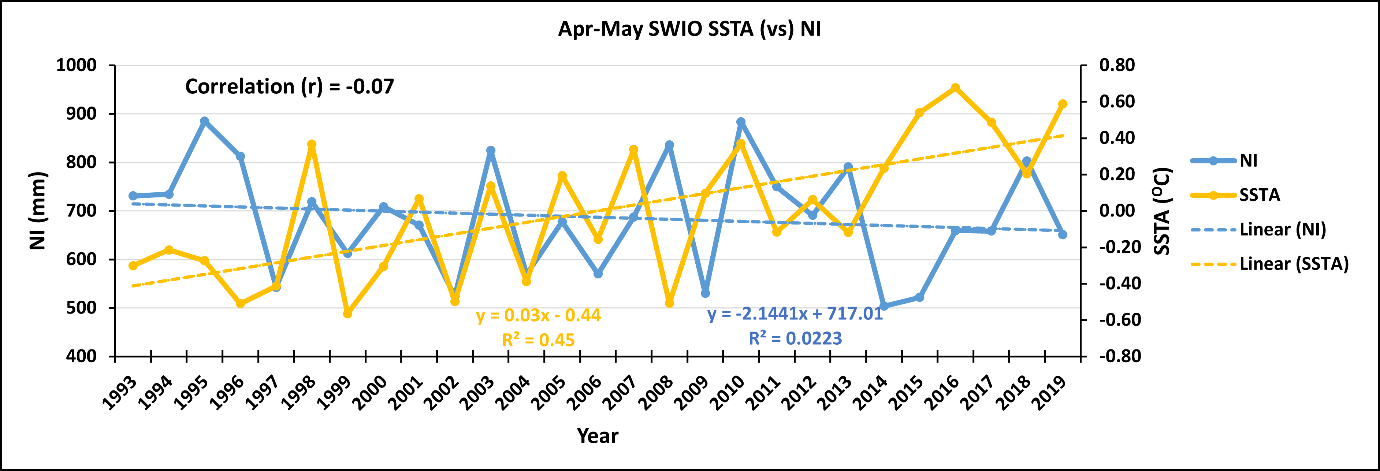


(d)


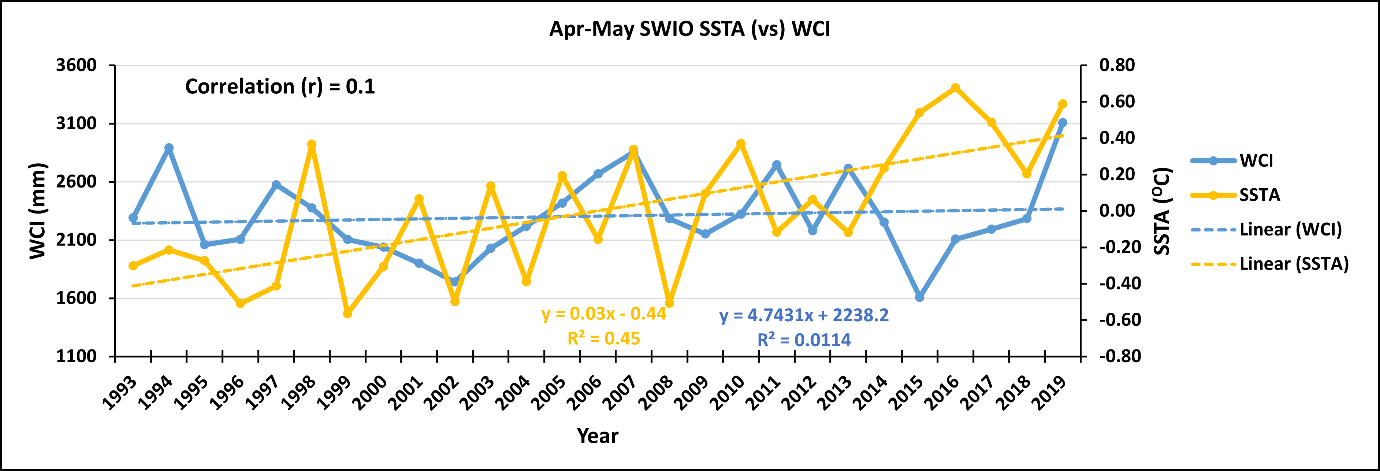


(e)

Figure S3: Time series of SWIO Apr-May SSTA and rainfall over homogeneous regions of (a) CI (b) EI (c) NE (d) NI and (e) WCI during 1993-2019. The figures are generated using Microsoft Excel.


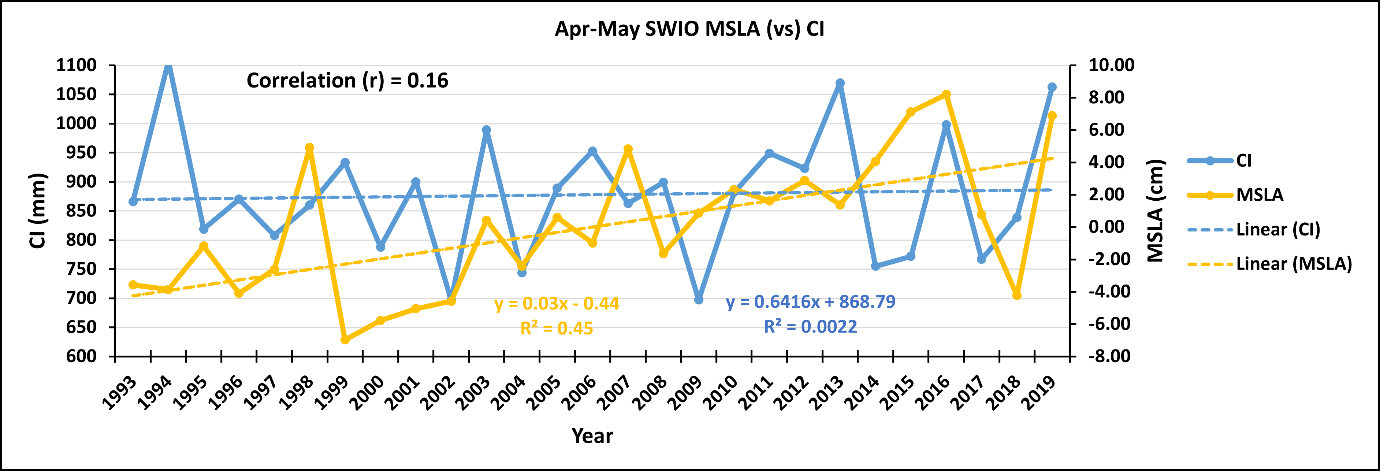


(a)


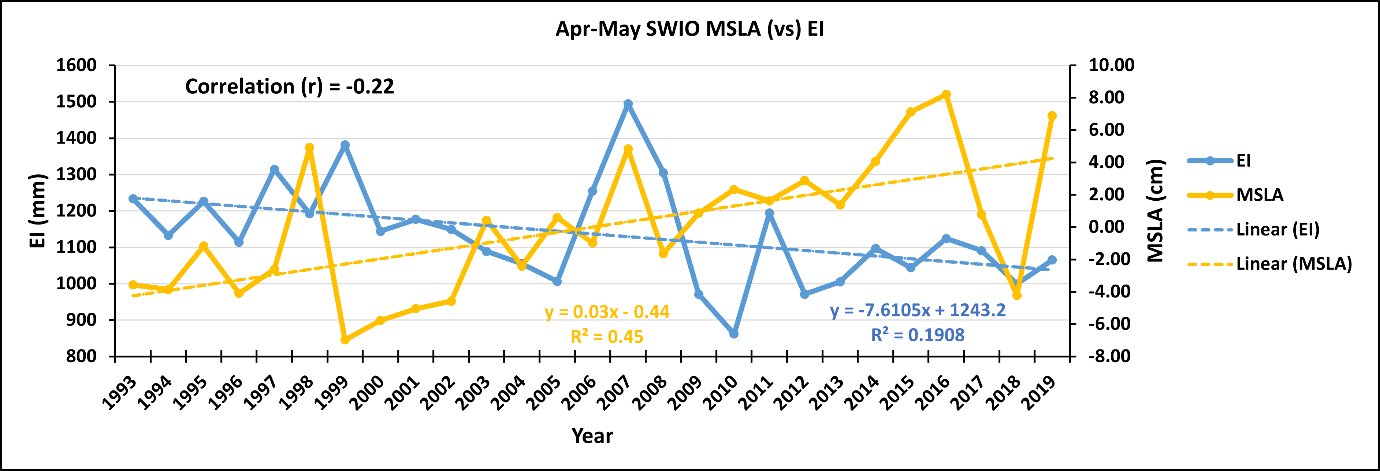


(b)


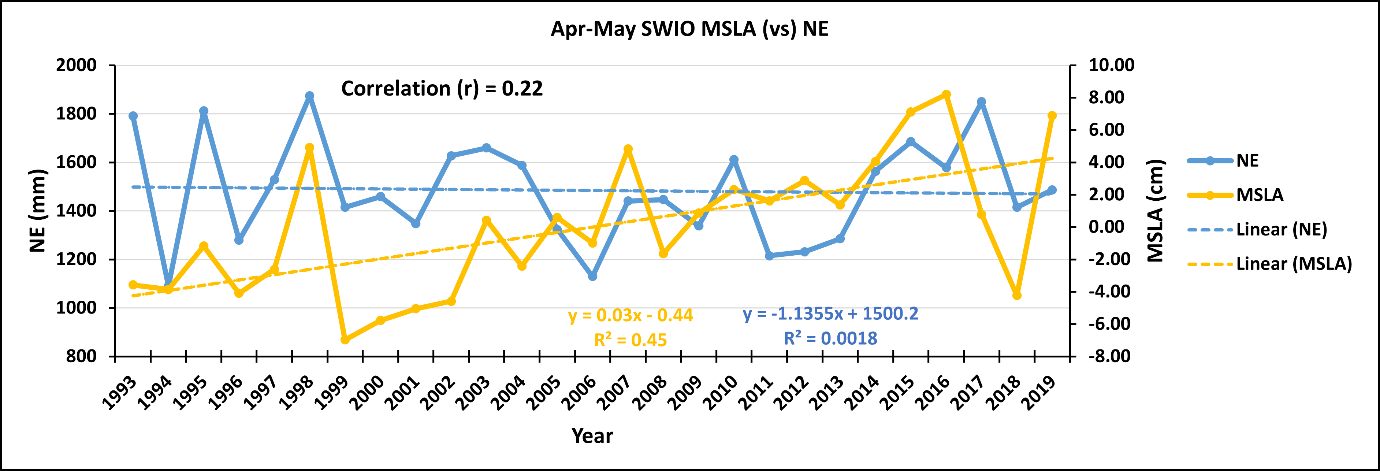


(c)


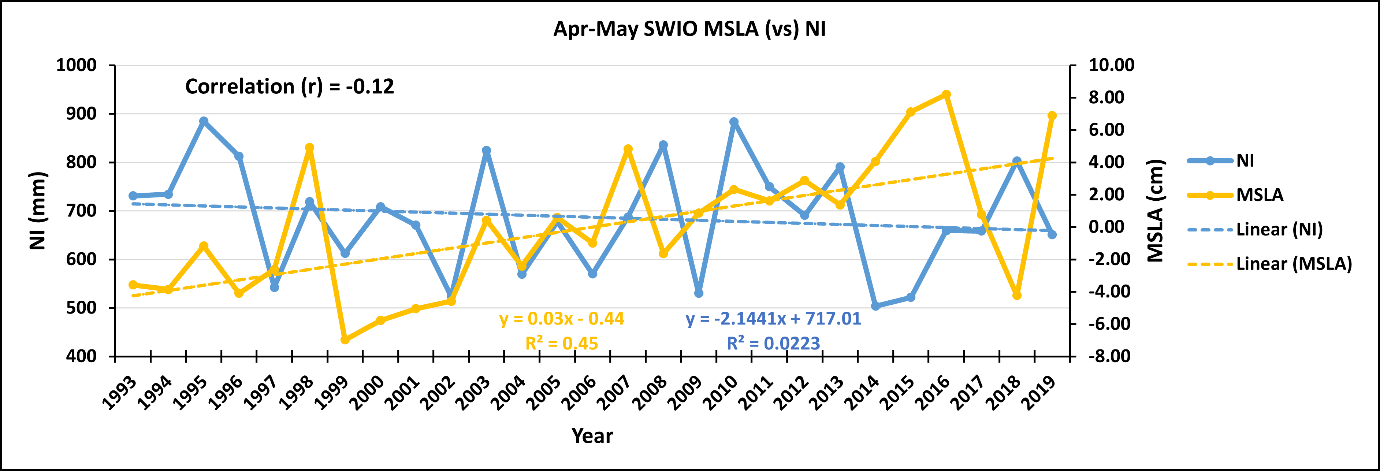


(d)


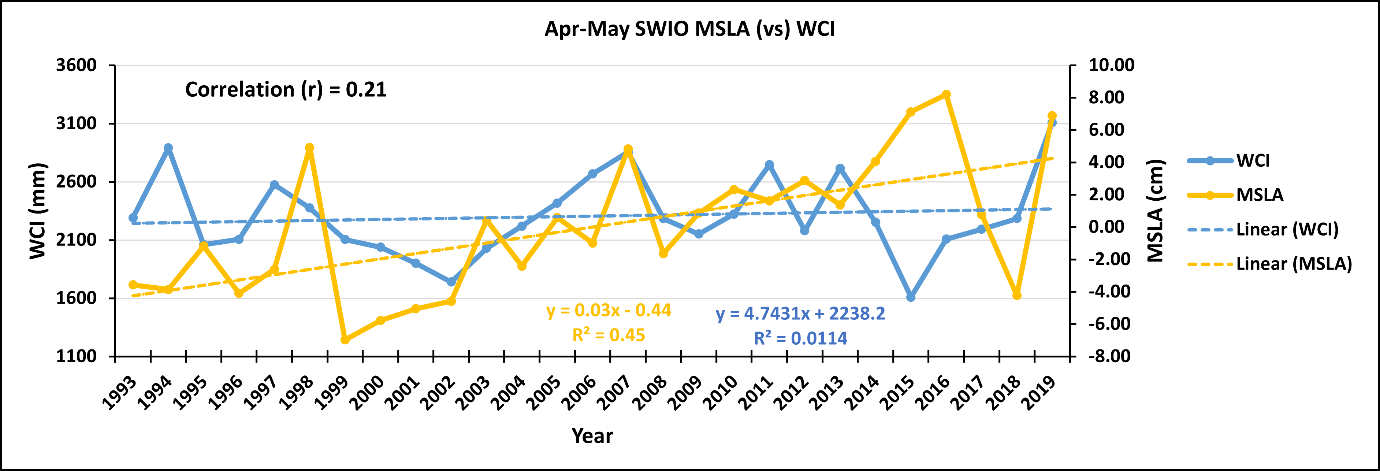


(e)

Figure S4: Time series of SWIO Apr-May MSLA and rainfall over homogeneous regions of (a) CI (b) EI (c) NE (d) NI and (e) WCI during 1993-2019. The figures are generated using Microsoft Excel.
